# Rbg1–Tma46 dimer structure reveals new functional domains and their role in polysome recruitment

**DOI:** 10.1093/nar/gks867

**Published:** 2012-09-23

**Authors:** Sandrea M. Francis, María-Eugenia Gas, Marie-Claire Daugeron, Jeronimo Bravo, Bertrand Séraphin

**Affiliations:** ^1^Instituto de Biomedicina de Valencia (IBV-CSIC), Calle Jaime Roig, 11, Valencia E-46010, Spain, ^2^Equipe Labellisée La Ligue, Institut de Génétique et de Biologie Moléculaire et Cellulaire (IGBMC), Institut National de Santé et de Recherche Médicale (INSERM) U964/Centre National de Recherche Scientifique (CNRS) UMR 7104/Université de Strasbourg, 67404 Illkirch, France, ^3^Equipe Labellisée La Ligue, Centre de Génétique Moléculaire (CGM), CNRS UPR3404, 1 Avenue de la Terrasse, 91198 Gif sur Yvette Cedex, France and ^4^Université Paris-Sud 11, 91405 Orsay, France

## Abstract

Developmentally Regulated GTP-binding (DRG) proteins are highly conserved GTPases that associate with DRG Family Regulatory Proteins (DFRP). The resulting complexes have recently been shown to participate in eukaryotic translation. The structure of the Rbg1 GTPase, a yeast DRG protein, in complex with the C-terminal region of its DFRP partner, Tma46, was solved by X-ray diffraction. These data reveal that DRG proteins are multimodular factors with three additional domains, helix–turn–helix (HTH), S5D2L and TGS, packing against the GTPase platform. Surprisingly, the S5D2L domain is inserted in the middle of the GTPase sequence. In contrast, the region of Tma46 interacting with Rbg1 adopts an extended conformation typical of intrinsically unstructured proteins and contacts the GTPase and TGS domains. Functional analyses demonstrate that the various domains of Rbg1, as well as Tma46, modulate the GTPase activity of Rbg1 and contribute to the function of these proteins *in vivo*. Dissecting the role of the different domains revealed that the Rbg1 TGS domain is essential for the recruitment of this factor in polysomes, supporting further the implication of these conserved factors in translation.

## INTRODUCTION

GTPases form a large family of universally represented proteins that have been involved in many cellular functions. Phylogenetic analyses have demonstrated that GTPases organize themselves in two distinct classes (1). The best-known branch was named TRAFAC as it contains GTPases involved in translation (TRAnslation FACtors). Besides translation factors, this branch also encompasses the well-known trimeric GTPases involved in signal transduction, septins and the RAS subfamily of GTPases. These proteins are characterized by the presence of a GTP-binding domain (G-domain) that contains five characteristic motifs, G1 [Walker A/P-loop, GxxxxGK(S/T)] responsible for binding of α- and β-phosphate groups of the nucleotide, G2 [Switch I, x(T/S)x] that binds Mg^2+^, G3 (Walker B/Switch II, DxxG) that interacts with the nucleotide γ-phosphate and Mg^2+^, G4 [(N/T)KxD] where K and D bind directly to the nucleotide, and the weakly conserved G5 involved in guanine base recognition. Small G-proteins have been extensively characterized and found to act as important molecular switches through changes in conformation related to the presence and nature of the bound nucleotide (none, GDP, GTP). In particular, the conformational changes occurring as a result of GTP hydrolysis has been shown to transduce cellular signals to downstream effectors mainly through changes in switch I (G2) and II (G3) regions (2). The critical function of GTPases in several biological processes is illustrated by the involvement of these proteins, and factors stimulating their catalytic activity or mediating nucleotide exchange, in many physiological disorders including cancer.

The TRAFAC class of GTPases is subdivided into several superfamilies (1). Among them, the classical translation factor subgroup contains the well-known family of ubiquitous translation factors (EF-Tu/EF-1α, EF-G/EF-2, initiation and termination factors) as well as three less well-characterized protein families named Bms1-like, HflX and OBG. Interestingly several of the latter factors were linked to ribosomes either through a direct role in translation or through their implication in ribosome biogenesis [e.g. (3,4–8)]. The Developmentally Regulated GTP-binding proteins (DRGs) belong to the OBG family of GTPases (which also contains the Obg/CgtA, YyaF/YchF, Nog1 and Ygr210 sub-branches). DRGs are strikingly conserved in archaea, fungi, plants and animals. In addition to the GTPase domain, these proteins also contain a C-terminal TGS domain of unknown function. TGS domains are also found in other GTPases of the OBG family and is shared with threonyl-tRNA synthetases (ThrRSs) and guanosine polyphosphate phosphohydrolases/synthetases (SpoT/RelA) [the acronym TGS being derived from ThrRS, GTPase and SpoT (9)]. DRGs were first characterized by their abundant expression in mouse embryonic brain showing subsequent downregulation in adult tissue (10,11). While archaea contains a single *drg* gene, two distinct DRG subtype, Drg1 and Drg2, are encoded by eukaryotic genomes (12). Some plants harbor three distinct genes, two of them code for nearly identical Drg2 subtype proteins that are likely to result from a recent gene duplication event (13). Two-hybrid screens and coimmunoprecipitation experiments revealed that DRG GTPases interact with conserved partner proteins in yeast and human. Those were named DRG Family Regulatory Protein (DFRP). Dfrp1 (also known as Lerepo4 in human) binds specifically to Drg1 while Dfrp2 preferentially binds to Drg2 (14,15). Dfrp1 and Dfrp2 contain a C-terminal region of ∼60 amino acids that was found to be required for binding to DRG and is named the dfrp domain (14). Else, Dfrp1 and Dfrp2 are highly divergent proteins, the former containing at its N-terminus two zinc fingers potentially mediating interactions with RNA while the latter contains a RWD domain that was identified in proteins interacting with the translational regulator Gcn1 (16). DFRP factor presence is important for the maintenance of normal levels of the cognate DRG proteins in human cells. Moreover, DRG–DFRP complexes were found to be localized in the cytoplasm of mammalian cells where the Drg1–Dfrp1 heterodimer was specifically found to associate with polysomes (17).

The yeast Drg1 homolog is named Ribosome-binding GTPase 1 (Rbg1) as it was found associated to ribosome (18,19). It associates with yeast Dfrp1, namely Tma46, which is also a ribosome-associated protein (15,18). Consequently, yeast Drg2 was named Rbg2 (Ribosome-binding GTPase 2) even if, like its human counterpart, it fails to cosediment with polysomes (15,17). Rbg2 associates with yeast Dfrp2, namely Gir2 (15). Consistent with the presence of a RWD domain, Gir2 was found to bind to Gcn1 (15,19). Yeast Rbg1 and Rbg2 are highly similar between themselves and with their human counterparts, Rbg1 sharing 66% identity and 80% similarity with human Drg1 and Rbg2 59% identity and 80% similarity with human Drg2. The sequence conservation of DFRP factors between these two species is however much lower.

Although phylogenetic evidence and biochemical fraction studies have linked the DRG proteins to translation, differentiation and growth, the exact molecular function of these GTPases is as yet unknown. Early studies have suggested that mouse and human Drg1 interacts *in vitro* and *in vivo* with the oncogenic T-cell acute lymphoblastic leukemia (Tal1/Scl) protein, a basic helix–loop–helix (bHLH) transcription factor involved in cell growth and differentiation (20,21). It was also reported that overexpression of Drg1 increased rat embryonic fibroblast transformation induced by c-myc and *ras* overexpression, affecting both the onset and average size of foci formed (20). Drg2 was also reported to be downregulated in SV-40 transformed fibroblasts in comparison to normal fibroblasts (22). In other studies, mammalian Drg1 was also found to be a target for SUMOylation stimulated by the MEKK1 Map3 kinase (23) or shown to interact with the protein kinase MPSK1 (STK16) in a process requiring the N-terminal 65 residues of Drg1 (24). In yeast, filamentous invasion into agar matrices by *Candida albicans* was attenuated by a Drg1 null mutation, concomitantly causing delayed lethality when the mutated organism was injected intravenously into mice. These phenotypes were suggested to result from the association of *C. albicans* Drg1 with Efg1 a bHLH transcription factor involved in repression of invasiveness (25). Many of these observations are difficult to reconcile with the conserved association of Drg1 factors to ribosomes. In yeast, deletion of *RBG1*, or *RBG2*, does not impair cell growth. Moreover, only very weak growth phenotypes resulting from double deletions of *RBG1* and *RBG2* could be detected using a sensitive competitive growth assay (26). An important step forward was made by the observation that a triple-deletion mutant lacking *RBG1*, *RBG2* and the gene encoding the putative RNA helicase Slh1 exhibited a strong negative growth phenotype (15). Importantly, translation is impaired in this triple mutant, as evidenced by the presence of reduced levels of polysomes. Similar phenotypes were observed for other combinations of mutation inactivating simultaneously the Rbg1–Tma46, Rbg2–Gir2 and Slh1 functions, suggesting that these three entities mediate overlapping functions in translation (15).

To gain further insights into the function of Rbg1 and Tma46 and the mode of interaction of these two proteins, we decided to investigate the structure of this heterodimer. Only a few structures of GTPase of the OBG subfamily are currently known. This includes *Bacillus subtilis* Obg (PDB ID 1LNZ) and human OLA1 of the YyaF/YchF subfamily (PDB ID 2OHF), the only structure available so far for a DRG subfamily member being the NMR solution structure of the C-terminal TGS of human Drg1 (PDB ID 2EKI). Our crystal structure revealed the presence of novel domains in Rbg1 and uncovered the mode of interaction of Rbg1 with Tma46. Based on this information, *in vitro* and *in vivo* assays allowed us to dissect the role of the Rbg1–Tma46 domains and interactions in GTPase function and polysome recruitment.

## MATERIALS AND METHODS

### Plasmids

Plasmids were constructed using standard cloning strategies or by site-directed mutagenesis using the QuickChange strategy (Stratagene, La Jolla, CA, USA) with minor modifications to manufacturer’s instructions. For expression constructs, an iterative trial and error process starting from plasmids encoding His6-tagged complete Rbg1 and full-length Tma46 was used. Protein yields, subunit interaction and homogeneity were assessed by gel electrophoresis after purification on Ni–NTA. When necessary, mass spectrometry analyses, apparent fragment sizes and sequence comparisons were used in an attempt to define suitable domain borders. For protein production, expression plasmids were transformed into BL21-CodonPlus (DE3)-RIPL. In some instances, glycerol stocks of the transformed bacteria were stored at −80°C until use. Material obtained from plasmids encoding stable and well-expressed products were tested for crystallization. Yeast plasmids were constructed as described above and contained genes expressed under the control of their native promoters. All constructs were verified by sequencing.

All plasmids used in this study are listed in Supplementary Table S1 while oligonucleotides used to prepare these constructs are listed in Supplementary Table S2.

### Purification of recombinant proteins

Protein expression was induced by growth in autoinduction media [Formedium (27)] plus kanamycin and chloramphenicol (50 µg/ml and 24 µg/ml, respectively) for 5 h at 37°C followed by overnight growth at 20°C. Cells were harvested by centrifugation and pellets kept frozen until further use. Rbg1fl–Tma46_205__–__345_ selenomethionine-substituted protein was obtained by using the autoinduction method (28,29). Small-scale protein production (100–200 ml) and purification were essentially performed as described earlier (30) except that BL21 CodonPlus was used for protein expression and buffer B (50 mM Tris–HCl pH 7.4, 20 mM imidazole, 300 mM NaCl, 2 mM ß-mercaptoethanol, 10% glycerol and 0.2% Igepal) for affinity purification on Ni–NTA. Proteins were eluted in buffer B containing 500 mM imidazole. For large-scale preparations, pellets were thawed on ice and mixed with lysis buffer [300 mM NaCl, 20 mM imidazole, 2 mM β-mercaptoethanol (β-MeOH), 50 mM Tris pH 8.0, 0.2% NP40 and a protease inhibitor cocktail tablet (Complete, EDTA-free, Roche)] and sonicated. After centrifugation at 30 600*g* at 4°C for 30 min, the lysate was filtered through a 0.45 μm sterile filter before loading onto a 5 ml HisTrap FF Chelating column pre-loaded with 100 mM NiSO_4_ and equilibrated in Buffer A [300 mM NaCl, 20 mM imidazole, 2 mM β-mercaptoethanol (β-MeOH), 50 mM Tris pH 8.0]. After washing the column with 10 bed volumes of Buffer A, proteins were eluted with a gradient of imidazole (20–500 mM) using an ÄKTA Purifier (GE Healthcare). Protein containing fractions were pooled and concentrated at 4°C to a final volume of 1–2 ml using Amicon Ultra centrifugal filter devices. The concentrate was directly loaded onto a pre-equilibrated size exclusion column [Sephadex 200 or 75 (16/60 or 26/60) columns] at 4°C and the protein eluted in buffer S (150 mM NaCl, 20 mM Tris pH 7.5 and 2 mM DTT) at rates of ∼ 1.0 ml/min using an ÄKTA Prime system. Purified proteins were then pooled and concentrated to 20–60 mg/ml by ultrafiltration at 4°C using the Amicon concentrator before flash-freezing in liquid nitrogen and storing at −80°C. The purification protocol for the selenomethionine-substituted proteins was as above except that in the last step of the purification 5 mM concentration of DTT was included to prevent selenomethionine oxidation.

### Crystallization, data collection, structure refinement and analysis

Crystals of Rbg1fl–Tma46_205__–__345_ complex were obtained by the vapor diffusion method. The drops were setup at 4°C with 1 μl of 60 mg/ml of protein and 1 μl of reservoir solution (2.38 M sodium formate, 0.2–0.5 M sodium citrate pH 6.5). Three dimensional rectangular crystals with typical dimensions 0.3 × 0.05 × 0.02 mm grew in about 2 weeks**.** The X-ray diffraction data for the native and selenomethionine derivative were collected from single crystals at the beam line ID14-4 (31) at the European Synchrotron Radiation Facility at Grenoble, France using an ADSC Quantum Q315r CCD detector.

The data were indexed and integrated using MOSFLM (iMOSFLM) and scaled with SCALA in the CCP4 suite (32). Heavy atom site search and phasing were done using SHARP (33) and model building/tracing were done using ARP/wARP (34). Cycles of manual model building were performed with the program Coot (35). Waters were introduced into the model using ARP/wARP program and validated with the electron density maps in Coot. The structures were refined with REFMAC (36) for isotropic refinement. TLS groups were defined and used for anisotropic refinement. This included 17 groups comprising of Rbg1 (chain A 2–45, 53–125/131–174/233–299, 175–232, 300–369; chain B 2–53, 54–91/98–125/133–174/233–299, 175–232, 300–369) and Tma46 (chain C 214–240, 241–267, 268–282, 302–313, 314–338; chain D 214–240, 241–267, 268–282, 320–336). Superimpositions between the structures were done using the SSM superpose function in Coot and analysis of the electrostatic surface potential was performed using APBS (37) in Pymol, also used for generating the structure figures (38).

### GTP binding and hydrolysis assay

Thermal shift assays were performed using protein samples at ∼0.05 mM in buffer S with 5 × Sypro Orange (Sigma), with or without 0.2 mM GDP, GTP or 0.5 mM GTPγS in wells of MicroAmp 96-well Fast Optical Reaction plate (Applied Biosystems). Fluorescence was measured from 20°C to 85°C in increments of 1°C in a T7500 Fast Real-Time PCR system (Applied Biosystems). Results were analyzed using the GraphPad Prism 4 software (GraphPad Software Inc.).

GTP hydrolysis assays using Malachite green (39) were performed as follows: solutions (5.72% w/v ammonium molybdate in 6 N HCl, 0.08% w/v malachite green solution, 2.32% w/v polyvinyl alcohol) were prepared individually using reagents from Sigma Aldrich and stored at 4°C. For assays, MilliQ water: Malachite green: polyvinyl alcohol: ammonium molybdate were mixed in a ratio of 2:2:1:1 and incubated for 3 h until they became yellow. Fifty microliters of protein samples (20 μM) in filtered and degassed buffer (100 mM KCl, 50 mM Tris pH 7.5 and 5 mM MgCl_2_) were incubated with GTP (Sigma Aldrich) for 1 h at 37°C in Microtest 96-well flat bottom plates (Sarstedt). An amount of 200 μl of Malachite green reagent was added in all wells and the absorbance readings at 630 nm were measured immediately in a Wallac Victor2 1420 Multilabel Counter. A phosphate standard prepared from KH_2_PO_4_ and blank with no protein were included on the plate. The latter background was subtracted from the protein sample readings. Data were fitted to Michaelis–Menten equation using non-linear regression in GraphPad Prism 4 to determine the kinetics.

### Yeast strains and growth assays

Yeast strains are all derived from BMA64 (40) and are listed in Supplementary Table S3. Strains containing a single disrupted and epitope-tagged gene were obtained by transformation with TAP-tag (41) and HISMX6 (42) modules carrying short flanking sequences homologous to the targeted gene. Primer sets that were used for that purpose are described in Supplementary Table S2. Transformants were checked for correct integration by PCRs. Plasmids were introduced into yeast strain using the standard LiOAc transformation method (43).

For growth assays, yeast cultures were grown to saturation in selective liquid media. The cultures were then diluted in water to an optical density at 600 nm (OD_600_) of 0.1. Three microliters of these cultures and 10-fold serial dilutions were spotted onto agar plates containing complete synthetic media minus leucine. Plates were incubated at 37°C and 30°C for 3 or 4 days, and cell growth was determined by visual inspection and documented by photography.

### Western blot analyses

Proteins from immunoprecipitation experiments, or total yeast extract (44), were fractionated by sodium dodecyl sulphate–polyacrylamide gel electrophoresis (SDS–PAGE) and subsequently transferred to nitrocellulose membrane. TAP-tagged proteins were detected as described earlier (45). HA-tagged proteins were detected using mouse anti-HA monoclonal antibody (Covance MMS-101P) and a secondary goat-anti-mouse IgG antibody (Jackson 115-035-068). As loading control Stm1 was detected by a polyclonal anti-Stm1 antibody and a secondary goat-anti-rabbit IgG antibody (Pierce 31460). Chemiluminescence was recorded with a LAS4000 device (GE Healthcare).

### Immunoprecipitation of epitope-tagged proteins

Logarithmically growing yeast cells in selective medium at 30°C were resuspended in lysis buffer containing 10 mM Tris–HCl pH 7.5, 150 mM NaCl, 5 mM MgCl_2_, 1 mM DTT and protease inhibitors. Cells were broken by mixing with glass beads. The cell extract was obtained by two consecutive centrifugations, the first 20 min at 14 000*g* and the second 10 min at 14 000*g*. Glycerol was added to a final concentration of 10%. An amount of 30 mg/ml of total proteins were incubated with IgG Sepharose Beads (GE Healthcare) or IgG coupled to Dynabeads on a rotor at 4°C for 2 h. Beads were pelleted and washed extensively with IPP150 buffer (10 mM Tris–HCl pH 7.5, 150 mM NaCl, 5 mM MgCl_2_). Proteins bound to the beads were eluted with SDS–PAGE sample buffer by boiling for 5 min.

### Polysome analyses

Polysomes were analyzed essentially as described previously (15).

## RESULTS

### Structure determination of the yeast Rbg1fl–Tma46_205__–__345_ complex

To obtain recombinant Rbg1 and Tma46, or truncated derivatives thereof, for structural and functional analyses, we constructed artificial operons encoding various versions of 6His-tagged Rbg1 followed by Tma46. Initially, full-length Rbg1 and Tma46 were used. The two proteins copurified indicating that no yeast-specific factor or compound was necessary to allow their interaction. However, low complex yields and truncated forms of Tma46 were observed. Mass spectrometry analyses and estimation of apparent molecular weights, together with sequence analyses delineating borders of conserved domains, provided rough estimates of the missing regions. After several iterative cycles of construct optimization, a plasmid expressing efficiently and without apparent degradation 6His-tagged full-length (fl) Rbg1 together with the C-terminal region of Tma46 (amino acids 205–345) encompassing the DFRP region was obtained.

The X-ray crystal structure of proteins obtained with the latter construct was solved to 2.67 Å resolution by the SIRAS method using a selenomethionine-substituted protein. The final model of the Rbg1fl–Tma46_205__–__345_ complex (*R*-factor 19.7%, *R*_free_ 22.2%) includes two molecules of the complex in the asymmetric unit although the complex behaves as a heterodimer by size-exclusion chromatography. The asymmetric unit contains Rbg1 molecules A and B interacting with Tma46 molecules C and D, respectively; molecule AC is used hereafter for the structural description. The data collection and refinement statistics are as given in Table 1. Rbg1 was modeled from 2 to 369 in both molecules A and B, but some of the loops, in particular those comprising the G-motifs, had poor electron density due to their flexibility. Seventy-seven water molecules in the first solvation shell were included.

**Table 1.** Data collection and refinement statistics of the Rbg1fl–Tma46_205–345_ complex structure

Data collection
Space group	P2_1_2_1_2	
Unit cell parameters		
a, b, c (Å)	86.2, 224.89, 84.89	
α, β, λ (°)	90.0, 90.0, 90.0	
	SeMet (peak)	Native
Wavelength (Å)	0.9795	1.0332
Completeness (%)	99.6 (99.6)	99.9 (99.9)
Mean I/σ(I)^a^	21.6 (5.9)	15.6 (4.4)
*R*_meas_ (%)^b^	8.3 (42.3)	8.1 (43.4)
Refinement		
Resolution (Å)		56.22–2.67 (2.74–2.67)
No. of reflections		46457
Reflections used in *R*_free_		1200
*R*_factor_^c^		19.7%
*R*_free_^d^		22.2%
Stereochemistry		
Res. in favored regions (%)		89.7
Res. in allowed regions (%)		10.2
Number of atoms		
Protein		7030
Water		77
Mean B-factor-Overall		76.935
RMSD^e^		
Bond lengths (Å)		0.007
Bond angles (°)		1.233
Residues modeled		
Rbg1 A		2–45, 53–125, 131–369
Rbg1 B		2–91, 98–125, 133–369
Tma46 C		214–282, 302–338
Tma46 D		214–282, 320–336
Residues with missing side chain		
Rbg1 A		Lys329
Rbg1 B		Ala46, Ser47, Ser48, Ser50, Lys369
Tma46 C		Glu307
Tma46 D		Leu214, Glu215, Asp320

Numbers in parenthesis indicate the highest resolution shell statistics. ^a^Mean [*I*/*σ*(*I*)] is the average of the relation between the intensity of the diffraction and the background.

^b^*R*_meas_ = {Σ*_hkl_* [*N*/(*N* − 1)]^1/2^ Σ_i_ |*I_i_*(*hkl*) − <*I*(*hkl*)>|}/Σ*_hkl_* Σ_i_ *I_i_*(*hkl*), where *I_i_*(*hkl*) are the observed intensities, <*I*(*hkl*)> are the average intensities and N is the multiplicity of reflection *hkl*.

^c^*R*_factor_ = Σ*_hkl_* {[*F_obs_*(*hkl*)] − [*F_calc_*(*hkl*)]}/Σ*_hkl_* [*F_obs_*(*hkl*)], where *F_obs_*(*hkl*) and *F_calc_*(*hkl*) are the structure factors observed and calculated, respectively.

^d^*R*_free_ corresponds to *R*_factor_ calculated using 2.5% of the total reflections selected randomly and excluded during refinement.

^e^RMSD is the root mean square deviation.

**Figure 1.**
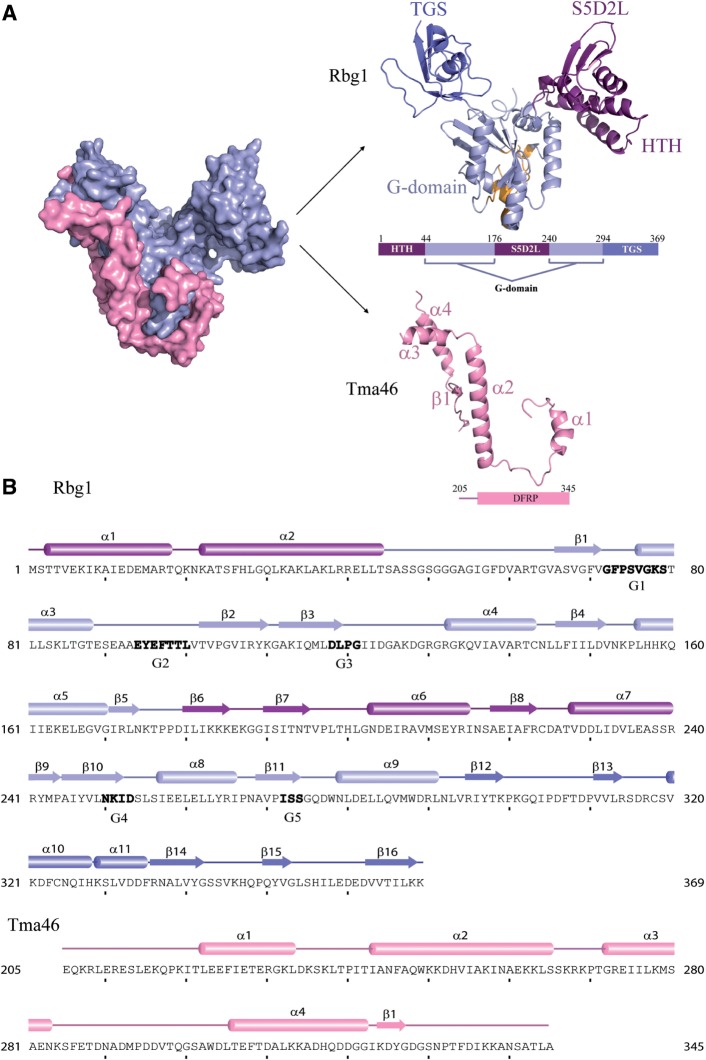
Structure of the Rbg1fl–Tma46_205–345_ complex with sequence information. (**A**) A surface representation of the Tma46 C-terminal fragment (pink) enveloping Rbg1 (pale blue) is shown on the left. The individual components are shown color-coded on the right: the Tma46 C-terminal fragment (pink) and Rbg1 with the G-domain (pale blue, this includes the short β sheet formed by β5 and β9 connecting the S5D2L domain), the protuberance formed by the HTH and S5D2L domains (purple) and the TGS domain (blue). The GTP-binding pocket is also represented with the five G motifs colored as orange. A schematic domain organization of the structurally solved complex is also shown. (**B**) The component sequences and secondary structure elements of the crystallized complex are represented with the G-motifs (G1–G5) given in bold letters. Domain boundaries are indicated in the same color scheme as in Figure 1A.

The Rbg1 structure shows a domain organization that includes the well-conserved G-domain (G1 + G2 + G3 = 64–169; G4 + G5 = 245–293), an N-terminal helix–turn–helix (HTH) domain (1–44) which lies adjacent to another domain formed by a 65-residue long insertion (176–240) between G3 and G4 of the G-domain and the TGS domain at the C-terminus (294–369) (Figure 1).

The G-domain of Rbg1 is highly similar to the well-conserved GTP-binding domain of other GTPases, in particular proteins Obg, YchF, FeoB, HflX, Ras-related proteins or Era, which all belong to the TRAFAC class of GTPases. The G-domain contains five α-helices (α3, α4, α5, α8 and α9) and six β-strands (β1, β2, β3, β4, β10 and β11). The G1 motif (P-loop/Walker A) is located on the loop connecting β1 to α3, G2 (Switch I) in between α3 and β2, G3 (Switch II/Walker B) at the end of β3 strand, G4 in β10 and G5 motif in β11 (Figure 1). While the G-domain of chain A and chain B adopt the same overall fold [root mean square deviation (RMSD) over the backbone Cα atoms is 0.96 Å], differences were observed especially in the loops containing the five G-motifs. Superimposing human OLA1 structure bound to ATP onto the G-domain of the two chains indicated that the P-loop in chain A adopted a closed conformation where entry of GTP could be difficult whereas chain B had an open conformation. The loops containing the G2 and G3 motifs were also shifted although the electron density in this area was not complete. We cannot however rule out that the observed conformational difference in this area might be due to crystal packing contacts.

Previous reports have shown that DRG factors contain about 65 amino acids inserted between the G3 and G4 motifs of the G-domain that are not found in other Obg family members and had no sequence homology to known domains (19). This region (residues 176–240) folds as an independent domain emerging from the G domain. We named this new domain of Rbg1 the S5D2L domain as database searches indicate that its topology is related to the ‘Ribosomal protein S5 domain 2-like’ (S5D2L) superfamily despite the absence of significant sequence similarity. The latter superfamily has 13 members (as according to Pfam, CATH and SCOP) and structural alignment shows that whereas the other members have a βββαβα fold, S5D2L domain has ββαβα fold lacking the first β strand (Supplementary Figure S1). The bacterial 30S ribosomal S5 subunit protein C-terminal domain is structurally the most similar to S5D2L domain, aligning with an RMSD of 2.2 Å over 51 residues although the sequence identity was very low (12%). Interestingly, the residues Gly and Arg of the S5 subunit protein known to cause ribosomal ambiguity when mutated (46) are fully conserved in Rbg1 (Gly189 and Arg207). Equivalent residues are also present in EF-G domain IV. The nature of these residues is however not universally conserved, as they are not found in GHMP kinase family members. A short parallel β sheet formed by β5 and β9 tether the S5D2L structure on the back of the G domain between the segments containing the G3 and G4 motifs.

The two amphipathic helices, α1 and α2 (2–44), comprises a previously unnoticed HTH domain at the N-terminal of Rbg1. While the HTH and S5D2L structures form a single globular protuberance emanating from the G domain, we refer to them as independent domains, as sequence phylogeny and functional data (see below) indicate that they behave as separate entities. The HTH is stabilized mainly by a hydrophobic zipper between the two helices, five leucines positioned 3–4 residues apart in the longer helix α2 contributing to the zipper while the other side of the helix α2 makes both hydrophilic and hydrophobic interactions with the S5D2L helices α6 and α7. Additionally, the interface between α1 and α2 is also stabilized by hydrogen bonds between Glu12 (α1)–Arg39 (α2) and Glu14 (α1) and His27 (α2).

The C-terminal TGS domain (294–369) has predominantly β sheet structure with five β strands (β12–β16) and two helices, α10 and α11 (Figure 1). Superimposition of the Rbg1 TGS domain with the previously reported NMR solution structure of human Drg1 TGS domain (PDB ID 2EKI) gave an RMSD of 1.31 Å over 76 Cα atoms. The TGS domain was found to be structurally very similar to threonyl tRNA synthetase, YchF and hOLA1 TGS domains and ubiquitin.

**Figure 2.**
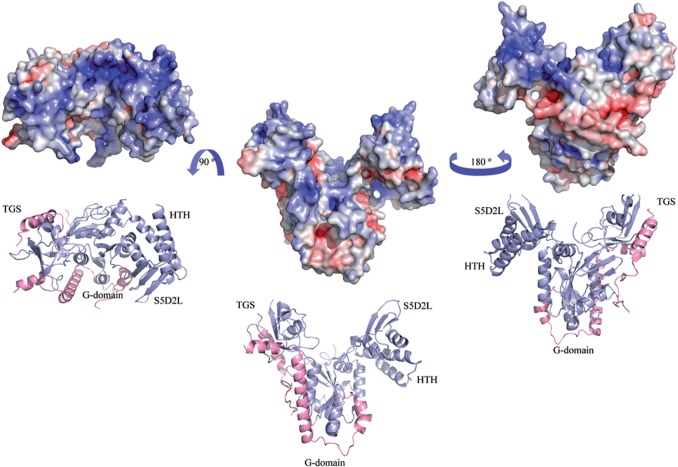
Electrostatic surface representation. The solvent-accessible surface electrostatic potential of the Rbg1fl–Tma46_205–345_ complex as calculated by APBS (Pymol) is shown as a surface alongside the cartoon representation. The potential is given with the negative (red) and positive (blue) contour levels in the range from −8.0 to +8.0 kBT respectively. The left figure shows the positively-charged surface formed partly by the G, HTH, S5D2L and TGS domains.

The HTH, S5D2L and TGS domains lie on the distal part of the GTP-binding pocket (Figures 1 and 2). Electrostatic surface potential analysis shows an extensive positively charged surface formed partly by the TGS, HTH, S5D2L and part of the G-domain opposite to the GTP-binding site (Figure 2).

### Tma46 structure and interaction with Rbg1

Tma46 fragment present in the structure shows a non-globular type of fold predominantly formed by α helices interconnected by coils. The residues of Tma46_205__–__345_ in the complex with Rbg1 that we could model into the electron density are located as mainly four helices (numbered in this study as α1–α4; note however that 19 residues between α3 and α4 were not modeled) and a short β strand which forms β sheet structure with adjacent β strands β2, β3, β1, β4, β10 and β11 from the G-domain of Rbg1. Tma46 helices envelops Rbg1 forming an extended and extensive interface (buried surface area in the interface ∼2978 Å^2^) contacting the G-domain (helices α1, α2 and ß strand) and the TGS domain (helices α3 and α4) with no contacts with the S5D2L and HTH domains (Figures 1 and 2). Based on weak sequence similarities between Dfrp1 and Dfrp2, it was earlier proposed that the region corresponding to residues 280–332 of Tma46 constituted a DFRP domain responsible for interaction with Rbg1 (14). Based on two-hybrid screen results, it was also previously suggested that Tma46 residues 254–296 would constitute the Rbg1-binding site (19). Our complex structure demonstrates that the region of Tma46 contacting Rbg1 is larger than these earlier estimates and encompasses residues 216–279 and 302–338. This observation suggests further that the DFRP region defined earlier is only a fraction of the biologically relevant unit involved in DFRP–DRG protein interaction and that the region of Gir2/Dfrp2 involved in contacting Rbg2/Drg2 is also larger.

One of the most prominent interacting surfaces between Rbg1 and Tma46 comprises α8 helix of Rbg1 (between G4 and G5 motifs), which inserts between helices α1 and α2 of Tma46 themselves interacting in turn also with Rbg1 α5 and α9 respectively. Helix α2 is longest in the fragment of Tma46 solved, and extends from the Rbg1 G-domain to the TGS domain that it contacts with its C-terminal end. Interestingly, the interface between Tma46 α2 and Rbg1 α8/α9 consists mainly of aromatic ring containing residues, which form a π-stacking interaction. Specifically, Tyr264 (α8) and Trp278 (α9) of Rbg1 form stacking interactions with Phe246, Trp249 and His253 of α2 of Tma46 (Supplementary Figure S2).

### Dissecting Tma46 interaction with Rbg1

These structural data allowed us to analyze in detail how Tma46 recognize Rbg1. As this involves a fragment of Tma46 that does not fold as a globular domain, but rather as a string of independent structural elements that meander on the surface of Rbg1, it is likely that Tma46 is intrinsically unfolded and only adopt the observed conformation upon binding to Rbg1. Interestingly, this is likely to also apply to Gir2 which was shown to be intrinsically unstructured (47).

**Figure 3.**
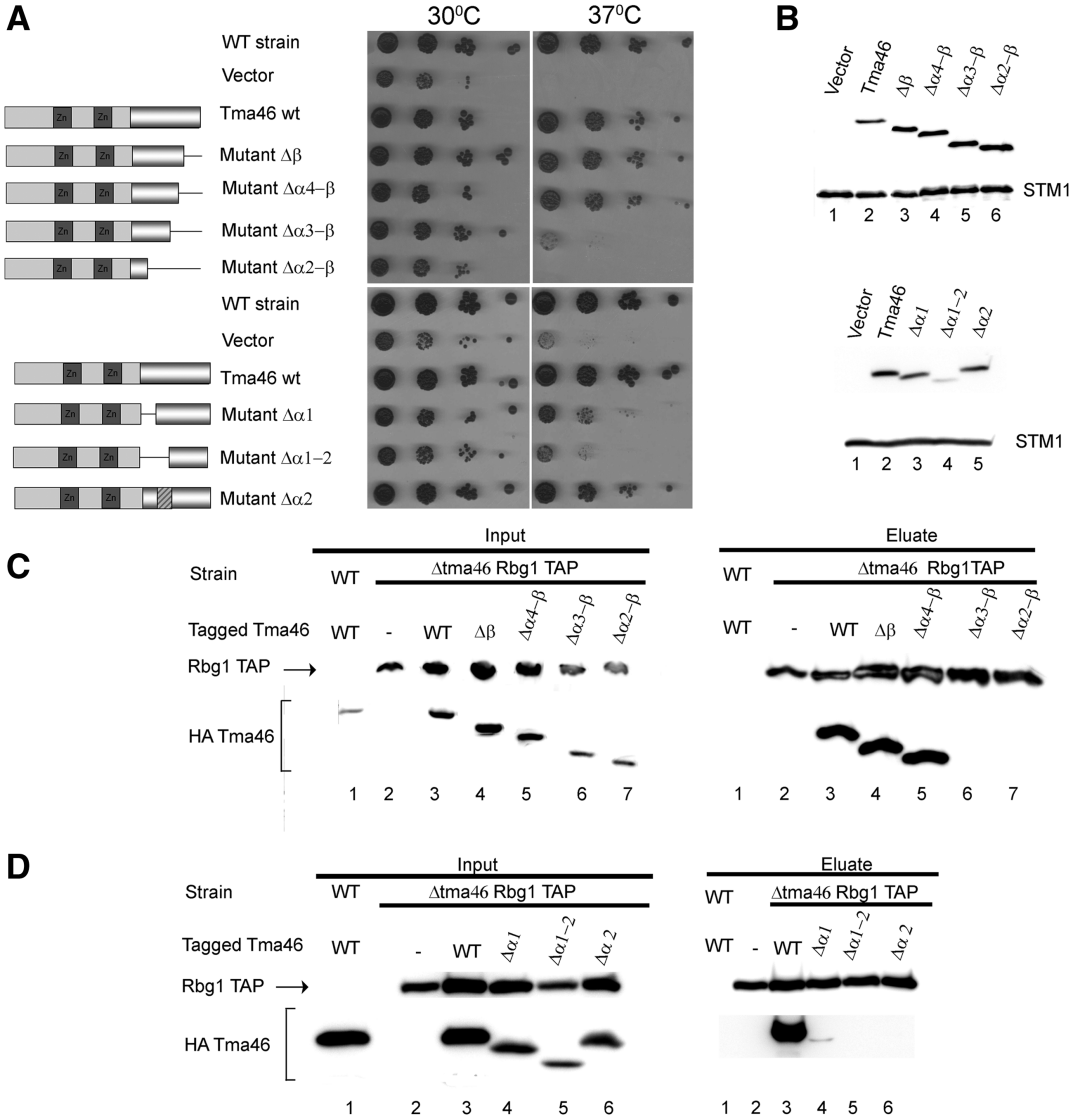
Analysis of Tma46 mutants. (**A**) Complementation assay for Tma46 function. The ability of plasmid-encoded Tma46 mutants to complement the growth phenotype of a triple *Δtma46Δgir2Δslh1* strain was assayed by spotting serial dilution on selective plates and incubating at 30°C or 37°C for 3 days. The structure of the various mutants is shown schematically on the left. Dark grey boxes indicate the Tma46 Zn-fingers, while the pseudo-cylinder represents the C-terminal region interacting with Rbg1. The hatched box indicates the alanine linker. WT strain indicates the original wild-type parental strain without mutation. (**B**) Mutant protein accumulation. The level of accumulation of the mutant proteins in cells shown on panel A grown at 30°C was assessed by detecting the HA tag by western blotting. Uniform loading is supported by analysis of the levels of the endogenous Stm1 protein. (**C**) Effect of C-terminal Tma46 truncation on its binding to Rbg1 in yeast. Extracts prepared from *Δtma46* strains carrying TAP-tagged Rbg1 and the various HA-Tma46 mutants grown at 30°C were used for immunoprecipitation on IgG beads. As control for the specificity of the coprecipitation a wild-type strain expressing wild-type Tma46 tagged with HA was used. Proteins present in extracts (Input) and (co)precipitated factors (Eluate) were analyzed by western blotting. (**D**) Effect of deletion of helices α1 and α1+α2 of Tma46 on binding to Rbg1. Samples were prepared as in panel C.

We constructed stepwise deletion of a HA-tagged version of the *TMA46* gene inserted in a yeast centromeric vector, removing defined structural elements that interact with Rbg1. Four mutants removing successively the Tma46 ß strand, helix α4, helix α3, helix α2 from the C-terminus and two mutants removing successively helix α1 and helix α2 from the N-terminus were built. Finally, we also constructed a mutant replacing helix α2 (residues 243–268) with an alanine linker of sufficient length to bridge helices α1 and α3. We first tested whether these mutants were functional by assaying their ability to complement the triple mutant *Δgir2Δtma46Δslh1* for its slow growth phenotype, a feature exacerbated at 37°C (15). We controlled by western blotting that the mutant proteins were expressed and accumulated to normal levels (Figure 3B). A control plasmid encoding a complete Tma46 restored a wild-type phenotype demonstrating that the presence of the tag does not impact on its function. Deletion of the Tma46 ß strand, alone or in combination with helix α4 does not impair Tma46 function. Removing the ß strand with helices α4 and α3 partially disrupts Tma46 activity while removing the region extending from the C-terminus and including helix α2 inactivate Tma46 (Figure 3A). Tma46 mutant levels were normal (Figure 3B) demonstrating that the partial activity did not result from protein instability but rather from inactivity. Reciprocally, deletion of helix α1, or replacement of helix α2 with an alanine linker, had limited effects on Tma46 level or function whereas deletion of helices α1 and α2 destabilized the protein resulting concomitantly in a poor complementation (Figure 3A and B).

We next assessed whether these mutations of Tma46 affect interaction with Rbg1 *in vivo*. For this purpose, plasmids encoding the mutant were introduced in a *Δtma46* strain carrying a TAP-tagged Rbg1 allele. Extracts prepared from transformants were incubated with IgG beads to precipitate TAP-tagged Rbg1 and associated factors. Presence of Tma46, and as a control of Rbg1-TAP, in the input and immunoprecipitated fractions (pellets) was assessed by western blotting (Figure 3C and D). This analysis demonstrated that deletions of the Tma46 ß strand or of the ß strand with helix α4, do not affect interaction with Rbg1. Only a low level of Tma46 lacking helix α1 was coprecipitated with Rbg1. In contrast, Tma46 proteins lacking larger fractions of the interaction region (deletion of ß strand with helices α4 and α3 or of ß strand with helices α4–2, substitution of helix α2 with a linker or removal of helices α1 and α2) do not coprecipitate with Rbg1 indicating that these mutations reduced affinity of Tma46 for Rbg1 or prevented interaction. Interestingly, overexpression of the latter mutants using high copy plasmids restored a specific interaction (Supplementary Figure S3) to a detectable level, indicating that all mutants are able to interact with Rbg1, albeit with much reduced affinity. Overexpressed Δα3–ß now complemented efficiently the *TMA46* deletion while the Δα2 construct complemented well independently of the vector used (Supplementary Figure S3). In contrast, the Δα2–ß and Δα1–α2 mutants were unable to rescue the Tma46 function even when overexpressed (allowing their accumulation at a level higher than Tma46 in the wild-type strain). Overall, these data suggest that interaction of Tma46 with Rbg1 is important to provide activity, even though we cannot formerly exclude Rbg-independent function of Tma46. More importantly, these data demonstrate that interaction of Rbg1 with Tma46 is not sufficient to provide activity. These results demonstrate further that none of the Tma46 elements (helices α1–α4 and ß strand) is absolutely essential for interaction and function. The presence of superfluous elements, demonstrated by the lack of functional phenotype and effect on interaction of several mutants, may ensure an extremely tight binding. Elimination of individual elements had different impact either as a result of different effect on affinity or because they contribute to additional function(s) beside interaction. The simultaneous removal of several elements always had stronger effect than removing individually these elements supporting the idea that they contribute in an additive manner to Rbg1 binding. Interestingly, increasing protein expression of interaction defective mutant was sufficient to restore Rbg1 binding suggesting that complex formation is controlled by the concentration of the two partners. However, even in such context, the larger deletions (Δα2–ß or Δα1–α2) were unable to complement a Tma46 deletion. This indicates that Tma46 is not simply sticking to Rbg1 but participates actively in the complex function, possibly by strengthening the contacts between the G and TGS domains of Rbg1 and/or by directly impacting on Rbg1 GTPase activity.

### GTP binding and hydrolytic activity of Rbg1 is modulated by interaction with Tma46

**Figure 4.**
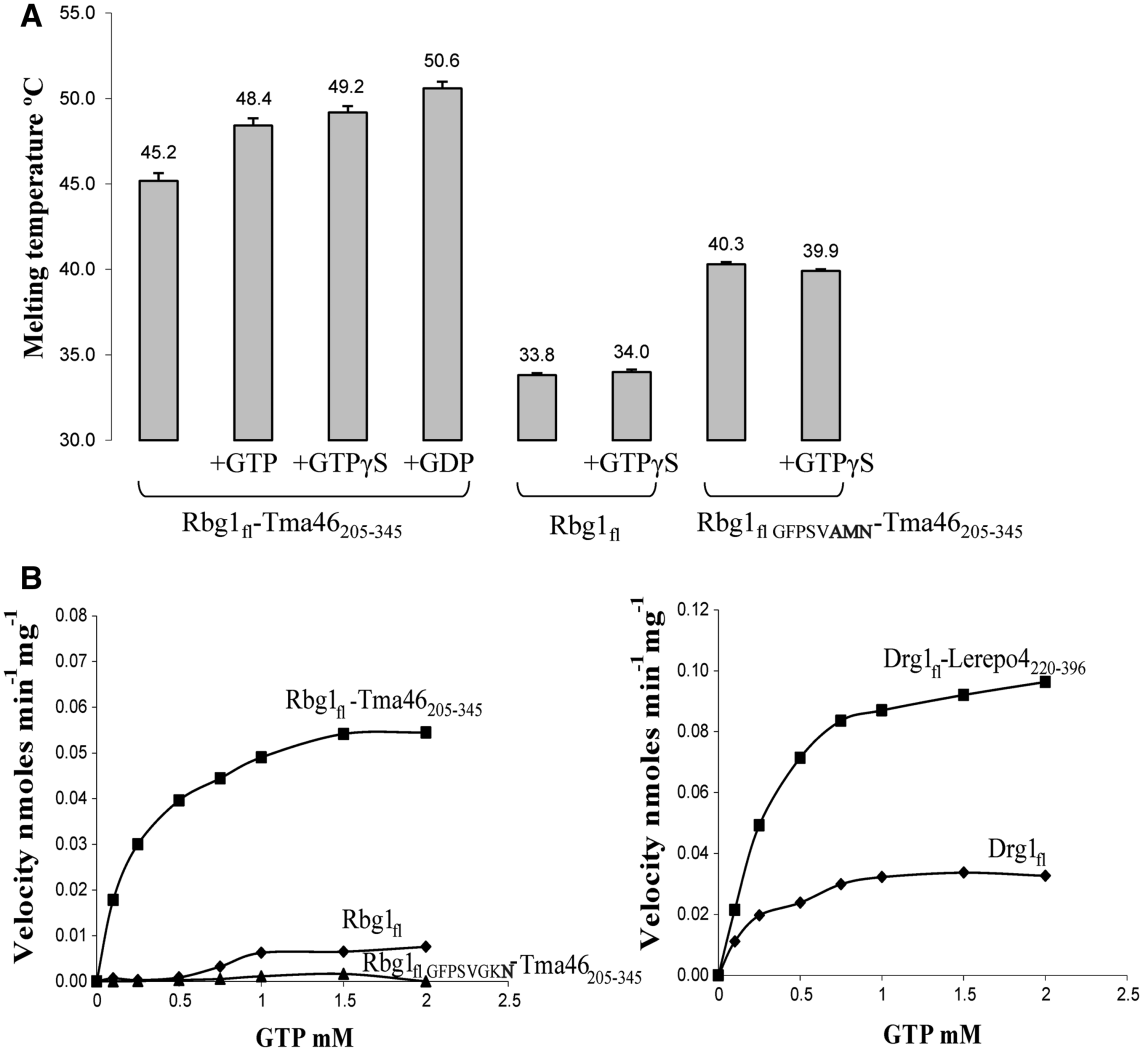
GTP binding and hydrolysis. (**A**) Presence of 0.2 mM GTP, GTPγS or GDP (in 4-fold excess over the protein concentration) causes an increase in melting temperature of the Rbg1fl–Tma46_205–345_ complex in the thermal-shift assay indicative of nucleotide binding. In contrast, addition of a 10-fold excess of GTPγS (0.5 mM) does not increase the melting temperature of Rbg1 alone. Also shown is the lack of increase in protein unfolding temperature for a Rbg1fl–Tma46_205–345_ G1 motif mutant (GFPSV**AMN**) in presence of 10× GTPγS. Note also that Rbg1_fl_ melts at a much lower temperature than Rbg1fl–Tma46_205–345_. (**B**) The GTP hydrolytic activity of Rbg1/Drg1, alone and in complex with Tma46_205–345_/Lerepo4_220–396_, respectively, is represented as a graph with increasing substrate concentration in the x axis. A catalytic mutant Rbg1_fl VFPSVGK_**_N_** in complex with Tma46_205–345_ was used as a negative control.

DRG proteins have been shown to bind GTP and GDP (11,48). Moreover, Arabidopsis DRGs have been reported to hydrolyze GTP into GDP *in vitro* without the help of GAPs or GEFs, unlike Ras-like proteins (13). To investigate the effect of complex formation on GTP binding by Rbg1, we performed thermal-shift assay in the presence or absence of GTP, GDP or GTPγS for Rbg1 and Rbg1 with the C-terminal part of Tma46 (Figure 4A). Nucleotide binding of the complex was evidenced by an increased melting temperature in the presence of the GDP, GTP or non-hydrolysable GTPγS. Moreover, comparison of free Rbg1 and the Rbg1fl–Tma46_205__–__345_ complex revealed that the increased stability detected in the presence of GTPγS was specific for the complex, suggesting that complex formation favors nucleotide binding. As negative control, Rbg1fl–Tma46_205__–__345_ containing three mutations in the G1 motif (GFPSVGKS to GFPSV**AMN**) was used. No increase in the unfolding temperature of this mutant protein was seen in the presence of nucleotide. Since this mutation was designed to abrogate nucleotide binding, this observation confirms that the shift in Tm observed for the wild-type Rbg1fl–Tma46_205__–__345_ complex is indeed due to the protein–nucleotide complex formation.

We next analyzed the GTP hydrolytic activity of free-, and Tma46-bound-, Rbg1. The Rbg1fl–Tma46_205__–__345_ complex shows a weak GTP hydrolysis activity (Figure 4B and Table 2), with parameters similar to those reported for other Obg family of proteins (49,50) and atDrg1/atDrg2a (13). A catalytic site mutant S79N (G1 motif changed from GFPSVGKS to GFPSVGK**N,** which was shown to be inactive *in vivo* (15) did not show significant hydrolytic activity above the detection limit of the assay (Figure 4B) confirming the specificity of the reaction. Additionally, the recombinant human homolog, Drg1_fl_–Lerepo4_220__–__396_ complex was about twice as active as the cognate yeast complex (Table 2). For comparison, Rbg1 and Drg1 showed significantly reduced GTPase activity on their own (Figure 4). The increased activity of the complex over free Rbg1/Drg1 could be attributed mostly to the fact that Rbg1 might be more stably folded in presence of Tma46. This is evidenced, for example, by the decrease in activity of Rbg1 in complex with a Tma46 mutant lacking the helix α1 (Table 2). Furthermore, mutants of residues of Tma46 in the interface between Rbg1 (helix α8 and α9) and Tma46 (helix α2 and nearby residues) such as Ile241, Phe246, Trp249 and Lys250, the latter two of which are involved in π-stacking, also show reduced activity. Mutants of Tma46 at these positions displayed slightly reduced complementation of the *TMA46* deletion (Supplementary Figure S4). Consistent with the analysis of the Δα2 deletion, these mutants interacted with Rbg1 as well as wild-type Tma46 (Supplementary Figure S4). Thus, we conclude that specific contacts between Tma46 and Rbg1 are important to stimulate the GTPase activity of the latter *in vitro* and impact on Rbg1–Tma46 function *in vivo*.

**Table 2.** Kinetic parameters for GTP hydrolysis of the Rbg1–Tma46 complex and the mutant proteins

	*V*_max_ (nmol min^−1 ^mg^−1^)	*K_m_* (μM)	*k*_cat_ (min^−1^)
Rbg1_fl_–Tma46_205–345_	0.0593 ± 0.0018	304.1 ± 32.5	0.0034
Drg1_fl_ + Lerepo4_220–426_	0.1154 ± 0.0037	319.5 ± 35.0	0.0074
Rbg1_1–294_–Tma46_205–345_ (ΔTGS)	0.0166 ± 0.0022	426.3 ± 165	0.0008
Rbg1_Δ175–243+Gly_–Tma46_205–345_ (ΔS5D2L)	0.0157 ± 0.0028	221.5 ± 159.2	0.0006
Rbg1_fl_–Tma46_239–345_ (Tma46Δα1)	0.0370 ± 0.0012	191.2 ± 26.6	0.0020
Rbg1_fl_–Tma46_205–345 I241A, F246A_	0.0288 ± 0.0021	428.9 ± 94.7	0.0017
Rbg1_fl_–Tma46_205–345 I241A_	0.0406 ± 0.0018	186.0 ± 34.5	0.0023
Rbg1_fl_–Tma46_205–345 W249A K250E_	0.0345 ± 0.0014	425.8 ± 52.6	0.0020

### Role(s) of the different Rbg1 domains

The structure of the Rbg1–Tma46 heterodimer indicates that Rbg1 comprises four domains. The GTPase domain is the largest of them forming a platform surrounded by protuberances corresponding to the HTH, S5D2L and TGS domains. Previous analyses indicated that the GTPase and TGS domains of Rbg1 were essential for its function (15). However, careful examination of the structural data now available indicates that the TGS deletion used extended into the GTPase domain. This situation was probably responsible for the instability of the mutant protein (15). The HTH and S5D2L had not been tested, as sequence analyses had not identified them unambiguously. We therefore constructed precise deletions of the HTH, S5D2L or TGS domains in a functional HA-tagged version of the *RBG1* gene inserted in a yeast vector. As deletion of the S5D2L removes an internal part of the protein, two mutants were built incorporating one or two glycine residues at the deletion point to allow sufficient flexibility and length of the polypeptide backbone to allow folding.

**Figure 5.**
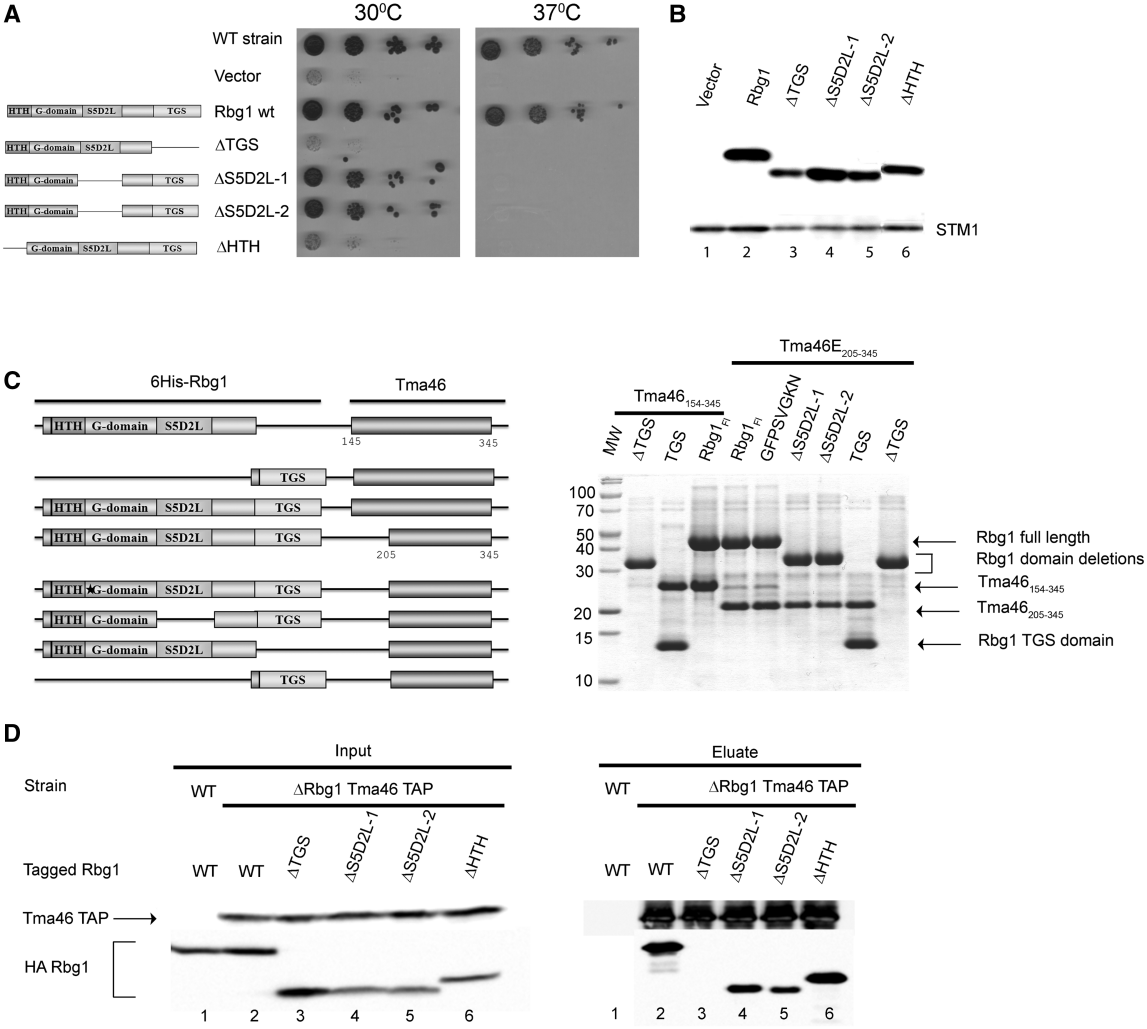
Analysis of Rbg1 domains. (**A**) Complementation assay for Rbg1 function. The ability of plasmid-encoded Rbg1 mutants to complement the growth phenotype of a triple *Δrbg1Δrbg2Δslh1* strain was assayed by spotting serial dilution on selective plates and incubating at 30°C or 37°C for 4 days. The structure of the various mutants is shown schematically on the left. WT strain indicates the original wild-type parental strain without mutation. (**B**) Mutant protein accumulation. The level of accumulation of the mutant proteins in cells shown on panel A grown at 30°C was assessed by detecting the HA tag by western blotting. Uniform loading is supported by analysis of the levels of the endogenous Stm1 protein. (**C**) Interaction between recombinant Tma46 and Rbg1 mutants. Plasmids harboring operons encoding His6-tagged Rbg1 [wild-type, point mutant (GFPSVGK**N**) or mutant deleted for specific domains] together with Tma46 (either amino-acids 154–345 or 205–345) were used to express protein in *E. coli*. Recombinant proteins purified on Ni–NTA agarose were detected by Coomassie staining. Organization of the different operons is shown on the left. (**D**) Effect of C-terminal Tma46 truncation on binding to Rbg1 in yeast. Extracts prepared from *Δrbg1* strains carrying TAP-tagged Tma46 and various HA-Rbg1 mutants grown at 30°C were used for immunoprecipitation on IgG beads. As control for the specificity of the coprecipitation a wild-type strain expressing wild-type Rbg1 tagged with HA was used. Proteins present in extracts (Input) and (co)precipitated factors (Eluate) were analyzed by western blotting.

These mutants were first assayed for their function through their ability to complement the slow and temperature sensitive growth of a *Δrbg1Δrbg2Δslh1* strain (Figure 5A). Deletion of the HTH and TGS domain inactivated Rbg1. Interestingly, removal of the S5D2L complemented efficiently the mutant strain at 30°C but was unable to do so at 37°C (Figure 5A) even though the protein was stably expressed at both temperature (data not shown). This result demonstrates that deletion of the S5D2L domain did not disrupt the protein but that the presence of the S5D2L domain is essential for the full activity of Rbg1. Western blot analysis confirmed that all protein were expressed at (or near) wild-type levels indicating that the HTH, S5D2L or TGS domains are required for Rbg1 activity rather than stability (Figure 5B).

We next analyzed the roles of the different Rbg1 domains in mediating interaction with Tma46. Plasmids encoding full-length or truncated versions of Rbg1 together with regions encompassing the C-terminus of Tma46 (residues 154–345 or 205–345) were constructed. After expression in *Escherichia coli*, the recombinant proteins were purified on Ni–NTA thanks to the presence of a 6His tag inserted at the N-terminus of the Rbg1 fragment and protein eluates were analyzed by denaturing gel electrophoresis and Coomassie staining (Figure 5C). This analysis demonstrated that an inactive GTPase (G1 motif GFPSVGK**N**, S79N) or a form of Rbg1 lacking the S5D2L still interacted with Tma46. In contrast, deletion of the TGS prevented Tma46–Rbg1 interaction (even though low residual binding was occasionally detected). Interestingly, the TGS domain by itself was sufficient to interact with Tma46. Unfortunately, the effect of deleting the HTH domain could not be addressed in this assay because the corresponding protein was poorly expressed in *E. coli*.

To analyze the implication of the different Rbg1 domains in interaction with Tma46 *in vivo*, we introduced the shuttle plasmids encoding HA-tagged truncated versions of Rbg1 in a yeast *Δrbg1* strain expressing Tma46–TAP. Coprecipitation of the two proteins was assayed as described above (Figure 3). The results of this experiment demonstrated that deletion of the HTH or S5D2L had no impact on (HTH), or only reduced slightly (S5D2L), the capacity of Rbg1 to bind Tma46 while no interaction was detected after removal of the TGS domain (Figure 5D).

Altogether, consistent with the structure of the complex, these results demonstrate that the HTH and S5D2L domains do not contribute to Tma46 binding. These data reveal, however, a critical role for the TGS domain in the association of Tma46 with Rbg1.

We also assayed the GTPase activity of deletion mutants of Rbg1 lacking the S5D2L, or TGS, domain coexpressed with the C-terminal region of Tma46 (Tma46_205__–__345_). All mutants displayed a significant GTP hydrolytic activity albeit with reduced rate compared to the wild-type protein (Table 2). This result indicates that these two peripheral domains of Rbg1 are not essential for GTPase activity although they may modulate catalysis.

### Tma46 recruits Rbg1 in polysomes

**Figure 6.**
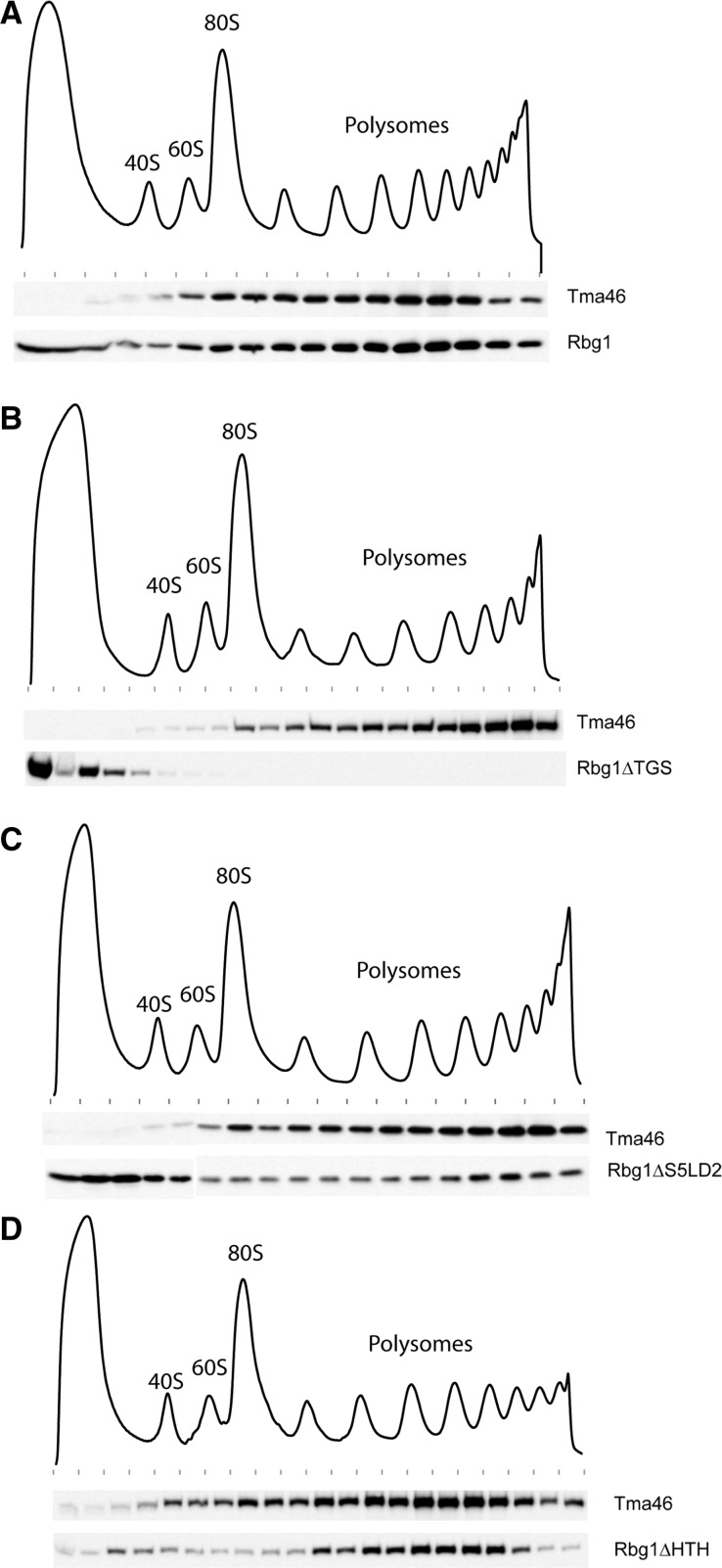
Polysome association of Rbg1 mutants. Polysomes extracts were prepared from cells expressing TAP-tagged Tma46 and HA-tagged Rbg1 (wild-type or domain deletion mutants). Polysomes were resolved by density sedimentation in 10–50% sucrose gradient. The UV absorbance trace (254 nm) obtained by continuous monitoring during fractionation is shown with the position of the 40S, 60S, 80S and polysomes peaks indicated. Fractions were analyzed by western blotting to detect the TAP and HA tags. (**A**) Distribution of Tma46-TAP and wild-type HA-Rbg1. (**B**) Distribution of Tma46-TAP and HA-Rbg1 ΔTGS. (**C**) Distribution of Tma46-TAP and HA-Rbg1 ΔS5D2L. (**D**) Distribution of Tma46-TAP and HA-Rbg1 ΔHTH. Previously reported control analyses demonstrate that Rbg1 association with polysome is specific (15).

Like their human homologs, Rbg1 and Tma46 have been shown to associate with polysomes (15,17,19). Thus, we next assessed the ability of Rbg1 deletion mutants to associate with polysomes. Extracts prepared from strains expressing wild-type TAP tagged Tma46 and the various mutant forms of Rbg1 tagged with an HA epitope were layered on sucrose gradients. After centrifugation, fractions of the gradients were collected while monitoring RNA absorbance (Figure 6). Presence of Tma46 and wild-type or mutant forms of Rbg1 in the various fractions was monitored by western blotting.

As reported earlier (15), wild-type Rbg1 and Tma46 cosediment in polysomes (Figure 6A). A similar distribution was observed when the HTH or S5D2L domains were deleted (Figure 6C and D), albeit increased free Rbg1 (consistent with a slightly reduced interaction with Tma46, see above) was detected at the top of the gradient in the latter case. Interestingly, removal of the TGS domain from Rbg1 resulted in complete segregation of the two proteins with Tma46 being localized in polysomes while Rbg1 remained at the top of the gradient (Figure 6B). Taken together with the observation that the TGS domain is sufficient and necessary to mediate interaction between Rbg1 and Tma46 (Figure 5), this observation is consistent with Tma46 mediating the recruitment of Rbg1 in polysomes.

## DISCUSSION

DRG proteins are extremely well-conserved factors present in eukaryotes and archaea that belong to the Obg/Drg GTPase subfamily (1). Characterized members of the latter group have all been implicated in ribosome genesis or function. Intriguingly, the two highly related DRG GTPases found in eukaryotes associate with rather distantly related DFRP cofactors, but still mediate partly overlapping functions.

To gain insights into the mode-of-action of these puzzling proteins, we determined the crystal structure of one of the yeast DRG, namely Rbg1, in complex with a fragment of its partner Tma46, and analyzed the functional roles of newly uncovered structural elements. Our results provide unexpected insights into the organization of DRG GTPases and into their mode of interaction with DFRP partners, including the impact of the latter on GTPase activity. Moreover, our data demonstrate a key role of Tma46 in mediating Rbg1 recruitment to polysomes. Sequence analyses suggest that a distantly related mechanism is exploited by the second eukaryotic DRG/DFRP pair (see below).

Sequence alignments had revealed the presence of GTPase and TGS domains in DRG proteins, including Rbg1. Moreover, the presence of an insertion implanted between conserved GTPase signature elements had been noticed (19). Our structure demonstrates that this insertion adopts a specific fold that protuberates on the surface of the Rbg1 GTPase module. This inserted domain, S5D2L, is related to domain 2 of the bacterial S5 protein. This ribosomal protein paves the entrance of the mRNA channel in the *E. coli* ribosome and is, interestingly, the target of mutations affecting translational fidelity (51,52). The presence of a related domain in a GTPase linked to ribosomal activity may thus be of functional significance. The structure of Rbg1 reveals in addition the presence of a HTH at the extreme N-terminus of the protein, whose presence had not been foreseen by sequence analyses. The HTH domain packs against the S5D2L while the TGS domain is distantly located in space and thus do not contact these two units. Overall, the Rbg1 protein, and related DRG factors, appear to be a multidomain GTPase with the GTPase core forming a platform on which are grafted three domains forming two independent protuberances (HTH and S5D2L on one side, and TGS on the other side). Mutational analysis indicates that the GTPase activity is absolutely required for Rbg1 function (15). Deletion of the HTH or TGS domain also abrogates Rbg1 activity, while, interestingly, removal of the S5D2L results in a conditionally active protein that is unable to function at high temperature. Thus, all domains of Rbg1 are required for its full activity. The S5D2L domain is not absolutely essential for function and may either stimulate protein activity under extreme conditions or be substituted by other factors in less demanding situations.

In the crystal structure, the C-terminal fragment of Tma46 adopts an extended conformation (Figure 1A) made essentially of successive α-helices interconnected by loops that meanders on the surface of the GTPase and TGS domains of Rbg1. Interestingly, part of Tma46 embraces an area of Rbg1 that is close to the GTP-binding site. Consistently, we observe that the presence of Tma46 affect GTP binding and hydrolysis by Rbg1. Tma46 may affect Rbg1 activity by strengthening contacts between the G and TGS domain and/or may modulate the GTPase activity through more subtle interactions. Deletion analyses indicate that the surface of interaction of Tma46 with Rbg1 can be reduced without abrogating binding or abolishing function, as long as a minimum is kept. Unexpectedly, all structural elements of this region of Tma46 are individually not essential, even if they contribute to binding and function. The region of Tma46 interacting with Rbg1 shows only limited conservation and is larger than the segment previously identified as the dfrp domain based on protein alignment. This part of Tma46 is unlikely to adopt a globular fold on its own, and thus probably corresponds to an intrinsically unstructured polypeptide. Accordingly, the Gir2 protein, that contains an equivalent region, has been shown to be intrinsically unstructured (47). Our results demonstrate that the C-terminal part of Tma46 contains structural elements superfluous for interaction with Rbg1 and function. Taken together with the lack of globular structure of this region and its extended conformation, it is tempting to propose that Tma46 evolved by the successive additions and extensions that increased its ability to interact with the Rbg1 surface. Such an evolutionary model is easy to imagine and fully consistent with the lack of obvious Tma46 or Gir2 homologs in archaea despite the presence of DRG homologs. Moreover, this framework also provide for an explanation for the poor sequence conservation of the Gir2 and Tma46 regions mediating interaction with Rbg2 and Rbg1 in yeast, and in the homologous proteins from other species, despite the extraordinary conservation of DRG factors. Indeed, any substitution arising in Dfrp protein still allowing efficient interaction with DRG factors will be accommodated, even if this results in a slightly altered relative structural arrangement. Overall, these constraints will result in an asymmetric rate of evolution of the two partners, with DRG factors changing slowly over time and DFRP proteins exploring rapidly an extensive sequence space. The availability of the Rbg1fl–Tma46_205__–__345_ structure might have provided explanation on why Rbg1 interacts with Tma46 *in vivo* while Rbg2 interacts with Gir2 despite the strong similarity between the two yeast DRG factors (15,17). However, mutagenesis of candidate residues failed to identify key amino acid interaction networks essential to establish this specificity (data not shown). This suggests that specificity is based on a large set of interactions rather than a few key amino acids. It is noteworthy also that the specific interaction of one DRG factor with a DFRP partner is not absolute, as cross-interaction can be detected in artificial conditions (19).

Contrasting with our observation that deletion of the Tma46 ß strand and helices α3 and α4 only reduces Rbg1–Tma46 interaction *in vivo* resulting in temperature sensitive function, deletion of the Rbg1 TGS domain abrogates the Rbg1–Tma46 interaction, both *in vivo* and in assays based on recombinant proteins. This observation is surprising because the region of Tma46 that interacts with the Rbg1 TGS domain is composed of helices α3 and α4. The latter result may indicate that the TGS domain contributes to Tma46 binding both by providing an extensive surface of contact for Tma46 and by maintaining the GTPase domain in a conformation favorable for interaction. Binding assays using recombinant factors confirm that Tma46 interacts efficiently with the isolated TGS while it binds inefficiently, at best, with a truncated form of Rbg1 lacking the TGS domain. These data support the idea that the stability of the Rbg1–Tma46 interaction resides for a major fraction in the area involving the TGS domain and/or that the TGS domain constitutes a primary nucleating center for the formation of this heterodimer. This demonstrate that the Rbg1 TGS domain has a critical role in mediating protein interaction and it will be of interest to test whether this property extends to TGS domains present in other proteins.

Interestingly, deletion of the TGS domain *in vivo* resulted in the fractionation of Tma46 and the mutant Rbg1 in distinct regions in polysome gradients: while Tma46 remained associated with polysomes, Rbg1 was released and found as a free factor in the lighter fractions of the gradient. This is a specific effect of the TGS deletion, as similar distributions were not observed with removal of the S5D2L or HTH domains of Rbg1. This suggests that Tma46 associates with polysomes, possibly through an interaction of its two Zn fingers with mRNAs and/or ribosomal RNAs, thereby recruiting Rbg1 in these assemblies. These observations strengthen the role of Rbg1 in translation and suggest that its GTPase would be able to mediate a yet-to-be-defined action on ribosomes after its recruitment. Interestingly, a variant of this model may apply to Rbg2 as well. Indeed, in specific conditions, an interaction of the N-terminal RWD domain of Gir2 with ribosome bound Gcn1 may provide a means to recruit Rbg2 to translating ribosome in a manner similar to the recruitment of Gcn2 (16). Further structural and biochemical work will be required to test the biological relevance of this hypothesis.

## ACCESSION NUMBERS

PDB ID code 4A9A.

## SUPPLEMENTARY DATA

Supplementary Data are available at NAR Online: Supplementary Tables 1–3, Supplementary Figures 1–4 and Supplementary References [15,40,53].

## FUNDING

Postdoctoral fellowship from the Ministerio de Ciencia e Innovación and Conselleria d’Educació, Generalitat Valenciana (to M.E.G.); Ministerio de Ciencia e Innovación [SAF2008-04048-E, SAF2009-10667 to J.B.]; Conselleria de Sanitat, Generalitat Valenciana [AP-001/10 and ACOMP/2012/039 to J.B.]; CSIC [200820I020 to J.B.]; European Union 6th Framework programs 3D Repertoire [contract LSHG-CT-2005-512028 to J.B. and B.S.]; CERBM-IGBMC (to B.S.); CNRS (to B.S.); Ligue Contre le Cancer [Equipe Labellisée 2011 to B.S.]. Funding for open access charge: CERBM GIE.

*Conflict of interest statement*. None declared.

## Supplementary Material

Supplementary Data
