# Supplementary material for: Rbg1–Tma46 dimer structure reveals new functional domains and their role in polysome recruitment
Source: Nucleic Acids Res. 2012 Sep 23;40(21):11100–14. doi: 10.1093/nar/gks867 (PMC3510508; doi:10.1093/nar/gks867)
Supplement: Supplementary Data [file supp_40_21_11100__index.html]

Rbg1–Tma46 dimer structure reveals new functional domains and their role in polysome recruitment — Rbg1–Tma46 dimer structure reveals new functional domains and their role in polysome recruitment — Supplementary Data 

# Rbg1–Tma46 dimer structure reveals new functional domains and their role in polysome recruitment

## Supplementary Data

files

**Files in this Data Supplement:**

- Supplementary Data - pdf file
