# Supplementary material for: Rbg1–Tma46 dimer structure reveals new functional domains and their role in polysome recruitment
Source: Nucleic Acids Res. 2012 Sep 23;40(21):11100–14. doi: 10.1093/nar/gks867 (PMC3510508; doi:10.1093/nar/gks867)
Supplement: Supplementary Data [file supp_gks867_nar-00543-m-2012-File008.pdf]

SUPPLEMENTARY DATA for

Rbg1-Tma46 dimer structure reveals new functional domains and their role in polysome recruitment

Sandrea M. Francis[1], María-Eugenia Gas[2], Marie-Claire Daugeron[3,4], Jeronimo Bravo[1], Bertrand Séraphin[2,3]

[1] Instituto de Biomedicina de Valencia (IBV-CSIC), Calle Jaime Roig, 11, Valencia E-46010, Spain

[2] Equipe Labellisée La Ligue, Institut de Génétique et de Biologie Moléculaire et Cellulaire (IGBMC), Institut National de Santé et de Recherche Médicale (INSERM) U964/Centre National de Recherche Scientifique (CNRS) UMR 7104/Université de Strasbourg, 67404 Illkirch, France.

3 Equipe Labellisée La Ligue, CGM, CNRS FRE3144, 1 Avenue de la Terrasse, 91198 Gif sur Yvette Cedex, France

[4]Univ Paris-Sud, Orsay, F-91405

Correspondence to: Jeronimo Bravo, Instituto de Biomedicina de Valencia (IBV), Calle Jaime Roig, 11, Valencia – 46010, España. Tel: +34 96 339 1760; Fax: +34 96 369 0800; E-mail:. jbravo@ibv.csic.es, and Bertrand Séraphin, IGBMC (Institut de Génétique et de Biologie Moléculaire et Cellulaire), Illkirch F-67400, France Tel +33 (0)3 88 65 33 36; Fax +33 (0)3 88 65 33 37; E-mail: seraphin@igbmc.fr.

Supplementary Table 1: Plasmid used in this study

Names	Features	References
pBS4230	YCplac111-HA-RBG1 (ARS-CEN-LEU2)	(1)
pBS4236	YCplac111-HA-RBG1$_{1-293}$	this study
BS4237	YCplac111-HA-RBG1$_{\Delta 175-243}$ with a glycine inserted	this study
pBS4254	YCplac111-HA-RBG1$_{\Delta 171-239}$ with two glycines inserted	this study
pBS4426	YCplac111-HA-RBG1$_{49-369}$	this study
pBS4231	pFL36-HA-TMA46 (ARS-CEN-LEU2)	(1)
pBS4247	pFL36-HA-TMA46$_{1-320}$	this study
pBS4248	pFL36-HA-TMA46$_{1-304}$	this study
pBS4249	pFL36-HA-TMA46$_{1-268}$	this study
pBS4257	pFL36-HA-TMA46$_{\Delta 238-345}$	this study
pBS4258	pFL36-HA-TMA46-$_{W249A,K250E}$	this study
pBS4419	pFL36-HA-TMA46$_{\Delta 215-238}$	this study
pBS4420	pFL36-HA-TMA46$_{\Delta 215-269}$	this study
pBS4448	pFL36-HA-TMA46-$_{I241A\ F246A}$	this study
pBS4447	pFL36-HA-TMA46-$_{I241W}$	this study
pBS4715	pFL36-HA-TMA46$_{I243-R268->26A}$	this study
pBS4711	pRS425- HA-TMA46$_{\Delta 215-269}$	this study
pBS4712	pRS425-HA-TMA46$_{1-268}$	this study
pBS4713	pRS425- HA-TMA46$_{\Delta 238-345}$	this study
pBS4716	pRS425- HA-TMA46$_{I243-R268->26A}$	this study
pBS3080	6His-RBG1$_{fl}$	this study
pBS3445	6His-RBG1$_{fl}$-TMA46$_{205-345}$	this study
pBS3446	6His-RBG1$_{fl}$-TMA46$_{154-345}$	this study
pBS3447	6His-RBG1$_{272-369}$-TMA46$_{205-345}$	this study
pBS3448	6His-RBG1$_{272-369}$-TMA46$_{154-345}$	this study
pBS3449	6His-RBG1$_{1-294}$-TMA46$_{205-345}$	this study
pBS3450	6His-RBG1$_{1-294}$-TMA46$_{154-345}$	this study
pBS4308	6His-RBG1$_{(S72VGKN79)}$-TMA46$_{205-345}$	this study
pBS4311	6His-RBG1$_{\Delta 175-243}$-TMA46$_{205-345}$	this study
pBS4312	6His-RBG1$_{\Delta 171-239}$-TMA46$_{205-345}$	this study
pBS4432	6His-RBG1$_{\Delta 1-48}$-TMA46$_{205-345}$	this study
pBS4443	6His-RBG1$_{(I241A\ F246A\ )}$-TMA46$_{205-345}$	this study
pBS4444	6His-RBG1$_{(I241W\ )}$-TMA46$_{205-345}$	this study
pBS4445	6His-RBG1$_{(W249A\ K250E\ )}$-TMA46$_{205-345}$	this study
pBS4714	6His-RBG1$_{(S72VAMN79)}$-TMA46$_{205-345}$	this study
hDrg1	6His-DRG1$_{fl}$[a]	this study
hLerepo4$_{dfrp}$	6His-LEREPO4$_{220-396}$[b]	this study
Rbg1$_{s5d2l}$	Rbg1$_{176-240}$[b]	this study

[a] Human DRG1 cloned into pET24 vector.

[b] LEREPO4$_{220-396}$ was cloned into a modified version of pET28 vector from the pBS3445 plasmid with addition of a cleavage site for PreScission protease enzyme (Obtained vector from Dr. Ramón Campos, (2)).

Supplementary Table 2: Oligonucleotide used in this study

Primers	Sequence 5'-3'
OBS256	GGAGGAAATGAATTGGCACGTAGCTCGTCGAACATGATGAACTGTGC GTACGCTGCAGGTCGAC
OBS257	TTATATATACGGTGGCATATATACAGTTTAGTCTATATGAATTTTAGAA TCGATGAATTCGAGCTCG
OBS264	GGTAGCAACCCCACCTTCGACATCAAAAAGCCAACTCCGCCACGCTC GCATCCATGGAAAAGAGAAG
OBS265	GTTGGTGTGTGTGCGTGTATTATATATACGGTGGCATATATACAGTTTA GTCTACGACTCACTATAGGG
OBS1111	TTTGAAGCTAGATGCAGTAGGTGCAAGCGTAGAGTTGTTGATTGAGCA AACGTACGCTGCAGGTCGAC
OBS1112	CCGATACCCCCTTTTCGAATACAAATGCTTGTATATTCAATTATGAATT ATCGATGAATTCGAGCTCG
OBS1118	GGTTGAGTCACATTTTGGAAGACGAAGATGTTGTTACCATCTTGAAAA AGTCCATGGAAAAGAGAAG
OBS1119	CCGATACCCCCTTTTCGAATACAAATGCTTGTATATTCAATTATGAATT ATACGACTCACTATAGGG
OBS2067	GTCGGTGGGGAAAAATACATTACTGTCC
OBS2068	GGACAGTAATGTATTTTTCCCCACCGAC
OBS3378	GGCTCTAGATAAGGAGGATATATATGCATCACCATCACCATCACATGT CTACTACAGTTGAAAAAA
OBS3379	GCCGGCGCGCCTCACTTTTTCAAGATGGTAA
OBS3380	GGCTCTAGATAAGGAGGATATATATGCATCACCATCACCATCACATGA TTTCGTCTGGTCAAGATTG
OBS3382	GCCGGCGCGCCTTAGACTAGATTTAGTCTATCCC
OBS3383	GGCGGCGCGCCTAAGGAGGATATATATGGAGCAAAAGAGGCTCGAGA G
OBS3384	GCCGAGCTCCTATGCGAGCGTGGCGGAGT
OBS3385	GGCGGCGCGCCTAAGGAGGATATATATGGGTAACCCAAAGACCACCA CC
OBS4716	TGTTCGAGAATCATAGATTTAGTCTATCCCACATGACTTG
OBS4717	AAATCTATGATTCTCGAACAAAAAATAATATAAGAAATG
OBS4718	AGATGGCAGGACCATTCAGACGAATCCCCACACCTTCCAGTTCC
OBS4719	TCGTCTGAATGGTCCTGCCATCTATGTGTTAAACAAGATTGATTC
OBS4720	TGTATCTTCTACCACCCCCCACACCTTCCAGTTCCTTCTCAATGATTTG
OBS4721	AGGTGTGGGGGGTGGTAGAAGATACATGCCTGCCATCTATGTGTTAAA C
OBS4722	GAATTTTAGACTAGTCGTCTTGGTGGTCAGCCTTCTTCAAG
OBS4723	AGACGACTAGTCTAAAATTCATATAGACTAAACTGTATATATG
OBS4724	GAATTTTAGACTAATCCCATGCGGATCCCTGGGTGACGTC
OBS4725	ATGGGATTAGTCTAAAATTCATATAGACTAAACTGTATATATG
OBS4726	GAATTTTAGACTACCTCTTACTAGATAACTTCTTTTCAGCG
OBS4727	TAAGAGGTAGTCTAAAATTCATATAGACTAAACTGTATATATG
OBS4730	AACAATTGCCAACTTCGCCCAAGCTGAAAAGGACCACGTCATCGCAA AG
OBS4731	CTTTGCGATGACGTGGTCCTTTTCAGCTTGGGCGAAGTTGGCAATTGTT
OBS4738	GAATTTTAGACTACTTGGATTTGTCTAGTTTCCCTCTTTC
OBS4739	ATCCAAGTAGTCTAAAATTCATATAGACTAAACTGTATATATG
OBS5211	AGCTAACACCAGCAACAATTGCCAACTTCGCCCAATG
OBS5112	TCACCATCACGGCAGCGGTGGTGGTGCTGGTATTG

OBS5113	CACCGCTGCCGTGATGGTGATGGTGATGCAT
OBS5114	GGAGTCGCTCACACCAATAACAATTGCCAACTTCGCCCAATG
OBS5115	TTATTGGTGTGAGCGACTCCCTCTCGAGCCTCTTTTGCTC
OBS5116	GGAGTCGCTCCCTACTGGTAGAGAAATCATTCTTAAGATGAG
OBS5117	TACCAGTAGGGAGCGACTCCCTCTCGAGCCTCTTTTGCTC
OBS5135	AGTCGACCATGGCAGCGGTGGTGGTGCTGGTATTGG
OBS5136	CACCGCTGCCATGGTCGACTCTAGAGGATCCTGCATAG
OBS5192	CCAACTTCGCCCAAGCGGAGAAGGACCACGTCATC
OBS5193	CTCCGCTTGGGCGAAGTTGGCAATTGTTATTGGTG
OBS5194	CTAACACCAGCAACAATTGCCAACGCCGCCCAATGGAAGAAGGACC
OBS5195	GGGCGGCGTTGGCAATTGTTGCTGGTGTTAGCTTGGATTTGTCTAGTTTCC
OBS5196	CTAACACCATGGACAATTGCCAACTTCGCCCAATG
OBS5197	GCAATTGTCCATGGTGTTAGCTTGGATTTGTCTAG
OBS5211	AGCTAACACCAGCAACAATTGCCAACTTCGCCCAATG
OBS5212	GGCAATTGTTGCTGGTGTTAGCTTGGATTTGTCTAG
OBS5213	AATTGCCAACGCCGCCCAATGGAAGAAGGACCACG
OBS5214	TCCATTGGGCGGCGTTGGCAATTGTTGCTGGTGTT
OBS5415	GCAGCCGCGGCGGCTGCAGCTGCAGCGGCTGCCGCCGCTGCGGCAGCTGTTATTGGTGTTAGCTTGGATTTGTCTAG
OBS5685	CCCAAGTCCATGGTTTGTGCTCTG
OBS5686	CAGCCGCCGCGGCTGCAGCTGCGGCAGCCGCCGCAGCGGCTGCCGCTAAACCTACTGGTAGAGAAATCATTCTTAAGATG
OBS5687	ATTAACGCGGCCGCGAATTCGCCCTTTTGTCGGGCG
OBS5749	GTAATGTATTCATCGCCACCGACGGGAACCCGACAAACCCCAC
OBS5750	GTCGGTGGCGATGAATACATTACTGTCCAAGTTGACTGGTACTGAGTC

Supplementary table 3: Yeast strains used in this study

Strains	Notes	Genotypes	Reference
BMA64α	WT (reference strains)	MATα, ade2-1 his3-11,15 leu2-3,112, Δtrp1, ura3-1 can 1-100	(3)
BSY1659	*RBG1::TAP Δtma46*	MAT?, Rbg1-TAP-$TRP1_{Kl}$, tma46::HISMX6	(1)
BSY1661	*TMA46::TAP Δrbg1*	MAT?, Tma46-TAP-$TRP1_{Kl}$, rbg1::HISMX6	(1)
BSY2049	*Δrbg1Δrbg2Δslh1*	MATa, Δrbg1:: HISMX6 Δrbg2:: kanMX4 Δslh1::TRPKl	(1)
BSY2057	Δ*tma46*Δ*gir2*Δ*slh1*	MATa, Δtma46::HISMX6 Δgir2::kanMX4 Δslh1::HISMX6	(1)

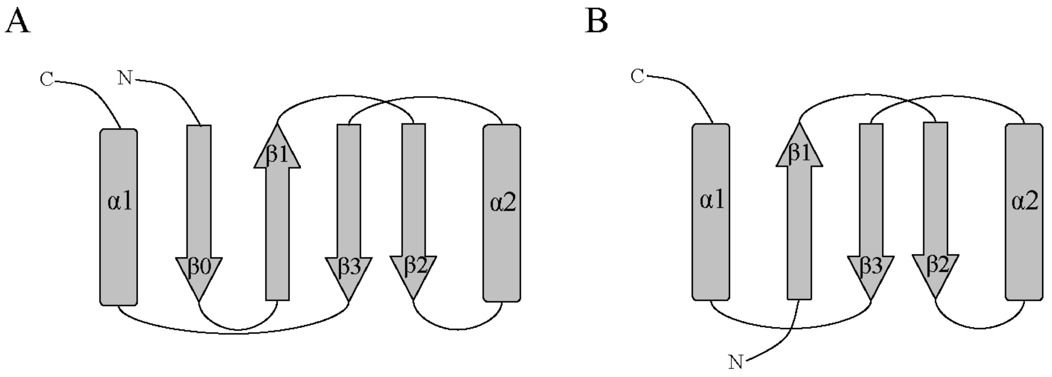

Supplementary Figure 1: A. The unusual βββαβα fold found in 30S and 40S S5 subunit protein C-terminal domain, Elongation factor domain IV, Mevalonate pyrophosphate decarboxylase (GHMP kinase family member) B. The S5D2L domain of Rbg1 having the ββαβα fold.

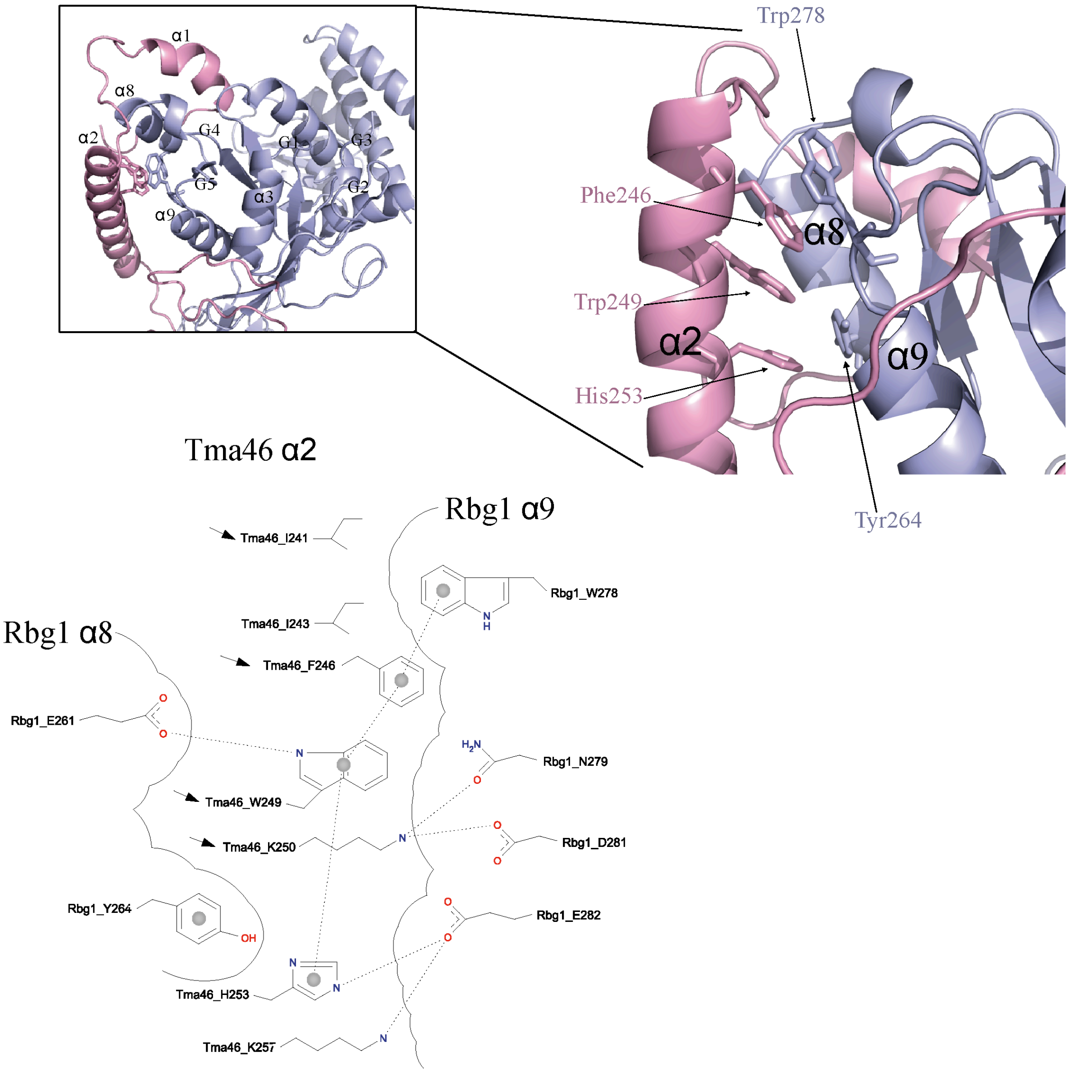

Supplementary Figure 2: Inset shows the region in the G-domain of Rbg1 where Tma46 helices α1 and α2 and surrounding residues bind. A zoom in on this interface shows, as stick representation, the aromatic residues whose rings stack over each other from Rbg1 helix α8/α9 and Tma46 helix α2 and nearby residues (helix α2 starts at position 243). Residues Trp278 from Rbg1 α9, Phe246, Trp249, His253 from Tma46 α2 and Tyr264 from Rbg1 α8 form the pi stacking interaction as can be seen in the two dimensional representation. The aromatic ring of Rbg1Tyr264 however interacts with its face to the Tma46 Trp249 and His253 edges in an offset position unlike the others, which form a staggered arrangement. The interface is additionally stabilized by the presence of electrostatic and hydrophobic residues.

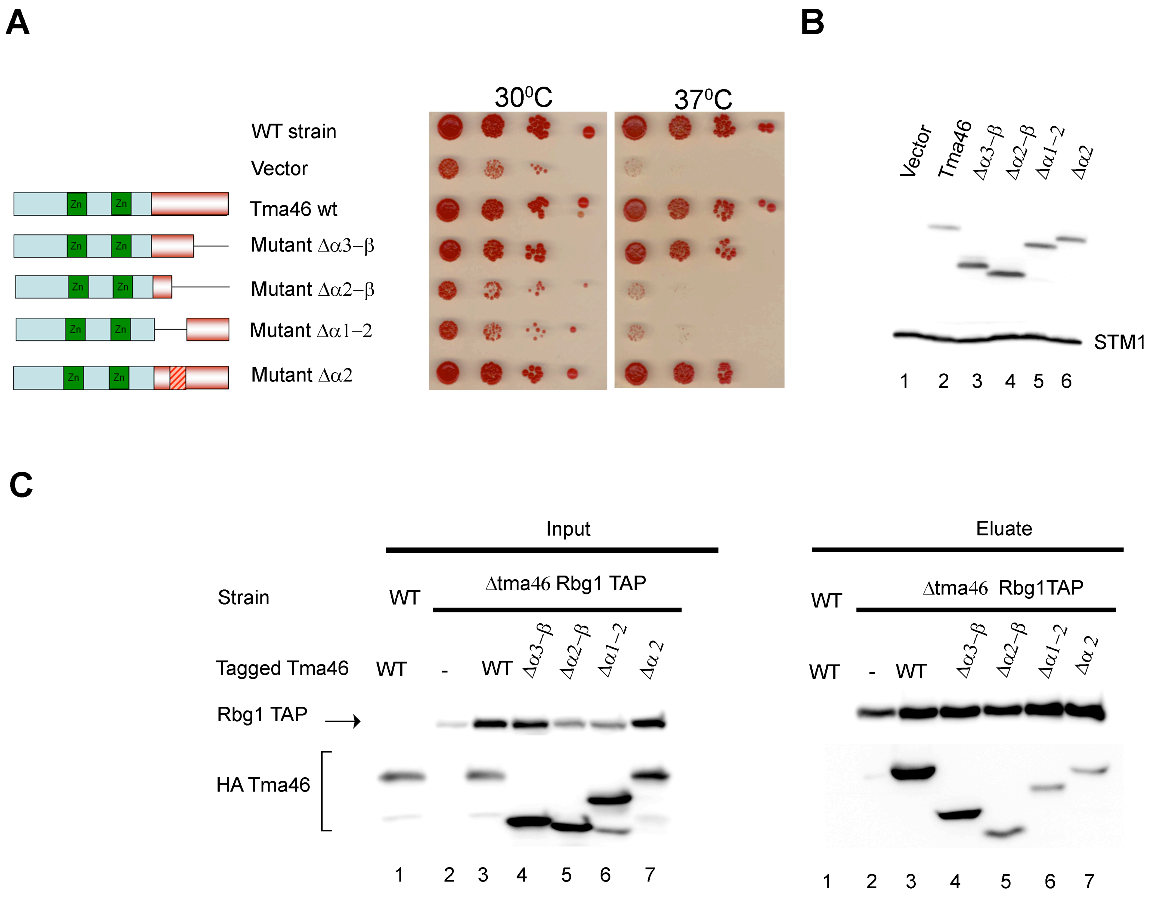

Supplementary Figure 3: Analysis of Tma46 mutant overexpressed in yeast

A. Complementation assay for Tma46 function. Tma46 mutants encoded on 2-micron plasmids were tested for their ability to complement the growth phenotype of a triple Δ*tma46*Δ*gir2*Δ*slh1* strain. Serial dilutions of the cells were spotted on selective plates and incubated at 30 or 37°C for 4 days. The structure of the various mutants is shown schematically on the left. WT strain indicates the original wild type parental strain without mutation. Tma46 wt indicates cells transformed with a centromeric plasmid encoding the wild type Tma46 protein. B. Mutant protein accumulation. The level of accumulation of the mutant proteins in cells shown on panel A grown at 30°C was assessed by detecting the HA tag by western blotting. Uniform loading is supported by analysis of the levels of the endogenous Stm1 protein. C. Binding of overexpressed Tma46 mutants carrying deletions of structural elements in its C-terminal region to Rbg1 in yeast. Extracts prepared from Δ*tma46* strains carrying TAP-tagged Rbg1 and the various HA-Tma46 mutants grown at 30°C were used for immunoprecipitation on IgG beads. As positive control, wild type HA-tagged Tma46 expressed from a centromeric plasmid was used. Specificity of the co-precipitation was assessed using a wild type strain expressing wild type HA-tagged Tma46 tagged. Proteins present in extracts (Input) and (co-)precipitated factors (Eluate) were analysed by western blotting. Analysis of the same Tma46 mutants expressed from centromeric plasmids is presented in Figure 3.

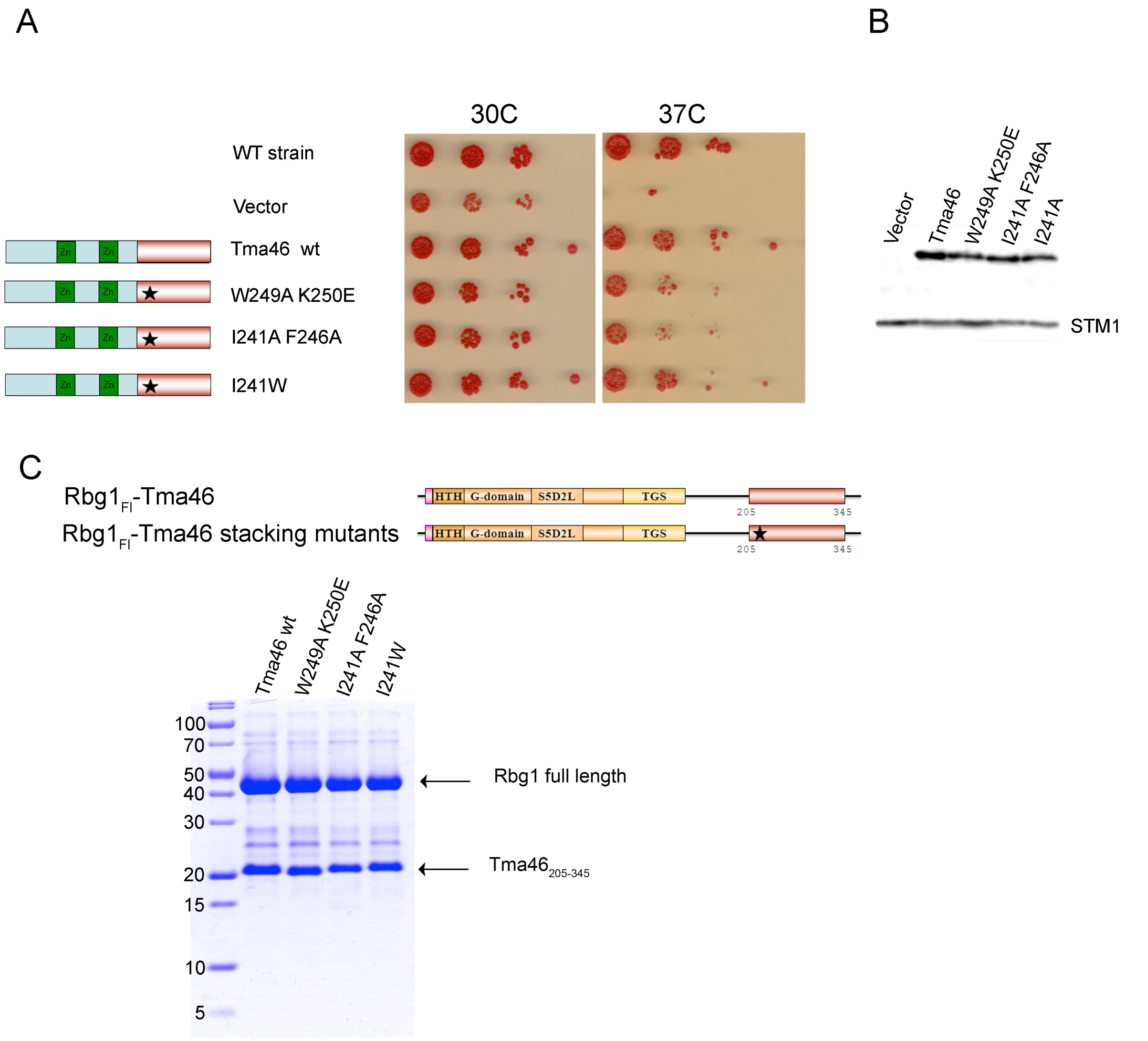

Supplementary Figure 4: Analysis of Tma46 point mutants at the interface of Rbg1 and Tma46

A. Complementation assay for Tma46 function. The ability of plasmid-encoded Tma46 mutants to complement the growth phenotype of a triple Δ*tma46*Δ*gir2*Δ*slh1* strain was assayed by spotting serial dilution on selective plates and incubating at 30 or 37°C for 3 days. The structure of the various mutants is shown schematically on the left. WT strain indicates the original wild type parental strain without mutation. B. Mutant protein accumulation. The level of accumulation of the mutant proteins in cells shown on panel A grown at 30°C was assessed by detecting the HA tag by western blotting. Uniform loading is supported by analysis of the levels of the endogenous Stm1 protein. C. Interaction between recombinant Rbg1 mutants and Tma46 point mutants. Plasmids harbouring operons encoding His6-tagged Rbg1$_{fl}$ together with wild type or mutant Tma46 (amino-acids 205-345) were used to express protein in *E. coli*. Recombinant proteins purified on NiNTA agarose were detected by Coomassie staining. Organization of the different operons is shown on the left.

References

1. Daugeron, M.C., Prouteau, M., Lacroute, F. and Seraphin, B. (2011) The highly conserved eukaryotic DRG factors are required for efficient translation in a manner redundant with the putative RNA helicase Slh1. *Nucleic Acids Res*, **39**, 2221-2233.
2. Campos-Olivas, R., Sanchez, R., Torres, D. and Blanco, F.J. (2007) Backbone assignment of the 98 kDa homotrimeric yeast PCNA ring. *J Biomol NMR*, **38**, 167.
3. Baudin-Baillieu, A., Guillemet, E., Cullin, C. and Lacroute, F. (1997) Construction of a yeast strain deleted for the TRP1 promoter and coding region that enhances the efficiency of the polymerase chain reaction-disruption method. *Yeast*, **13**, 353-356.
